# Frequent GU wobble pairings reduce translation efficiency in *Plasmodium falciparum*

**DOI:** 10.1038/s41598-017-00801-9

**Published:** 2017-04-07

**Authors:** Sherwin Chan, Jun-Hong Ch’ng, Mats Wahlgren, Jessada Thutkawkorapin

**Affiliations:** 1 Department of Microbiology, Tumor and Cell Biology (MTC), Karolinska Institutet, Box 280, Nobels väg 16, 171 65 Stockholm, Sweden; 2 Department of Microbiology and Immunology, National University of Singapore, Singapore, Singapore; 3 Department of Molecular Medicine and Surgery, Karolinska Institutet, Stockholm, Sweden

## Abstract

*Plasmodium falciparum* genome has 81% A+T content. This nucleotide bias leads to extreme codon usage bias and culminates in frequent insertion of asparagine homorepeats in the proteome. Using recodonized GFP sequences, we show that codons decoded via G:U wobble pairing are suboptimal codons that are negatively associated to protein translation efficiency. Despite this, one third of all codons in the genome are GU wobble codons, suggesting that codon usage in *P*. *falciparum* has not been driven to maximize translation efficiency, but may have evolved as translational regulatory mechanism. Particularly, asparagine homorepeats are generally encoded by locally clustered GU wobble AAT codons, we demonstrated that this GU wobble-rich codon context is the determining factor that causes reduction of protein level. Moreover, insertion of clustered AAT codons also causes destabilization of the transcripts. Interestingly, more frequent asparagine homorepeats insertion is seen in single-exon genes, suggesting transcripts of these genes may have been programmed for rapid mRNA decay to compensate for the inefficiency of mRNA surveillance regulation on intronless genes. To our knowledge, this is the first study that addresses *P*. *falciparum* codon usage *in vitro* and provides new insights on translational regulation and genome evolution of this parasite.

## Introduction

Degeneracy of the universal genetic code dictates that the 20 amino acids are decoded by 61 triplet codons. With the exception of methionine and tryptophan, any amino acid can be decoded by two to six synonymous codons (two – six fold degeneracy). In most organisms, the usage of synonymous codon is biased and drastic variations are observed between organisms. Mutational bias and selection force have been proposed to explain this phenomenon. The former suggests that mutational pressure acts on all DNA sequence (including codon sequence) and is strongly correlated to the species-specific genomic G+C content in both coding and non-coding region^1–4^. Yet, clear variations in codon bias can also be seen between genes within the same genome, suggesting strong selection force acting on the efficiency and accuracy of translation^5–7^. Importantly, these two theories are not mutually exclusive and both can influence and fine-tune codon bias.

During translation, codons are read by transfer RNAs (tRNA) that bear the matching anticodons. Specific base pairings between codons and anticodons allow amino acids to be incorporated into the nascent polypeptide correctly. However, the number of tRNA species is fewer than the 61 sense codons. Decoding of all codons is made possible by wobble base pairing^8^. While the first and second positions on a codon are subjected to strict Watson-Crick pairings with the anticodon, the third nucleotide of a codon can engage in non-standard pairing with the first nucleotide of the anticodon, and both are referred as the wobble positions. Wobble position on a tRNA can be modified to expand pairing capacity. For example, deamination of adenosine to inosine in ANN anticodon is permissive to wobble pairings (I:U, I:C, I:A), and is conserved in all eukaryotes^9^. Another common pairing is G:U/U:G wobbling. The U ending codons in all C/U ending two-box codon families (i.e. Asp, Asn, Cys, His, Phe and Tyr) are usually read by the corresponding GNN anticodons through GU wobble pairings. The ANN anticodons in these codon families are absent in most organisms and is believed to be important for preventing the mis-incorporation of incorrect amino acid through I:A pairing. The cooperative nature of codons and anticodons during translation imparts possible influence wielded by tRNAs on codon usage. Indeed, tRNA gene copy numbers do correlate with the abundances of the corresponding codon in some unicellular organisms^10, 11^, giving an extra weight to the selection theory.

Despite being unicellular organisms, all *Plasmodium* species only harbor a set of 45 non-redundant, nuclear-encoded tRNA isoacceptors, and there is no correlation between tRNA gene copy numbers and codon usages. Furthermore, the *Plasmodium* species sequenced so far demonstrate a wide range of genomic G+C content^12^, making it an interesting genus to interrogate in terms of codon bias. In particular, *P*. *falciparum*, responsible for most lethal episodes of malaria, has 81% A+T content in its genome, which is one of the highest of all sequenced genomes^13^. This extremity cumulates in the presentation of frequent insertion of asparagine homorepeats that have highly skewed A+T content in its codons (AAT and AAC). Amino acid repeats usually increase the propensity for protein aggregation, so the expansion of asparagine homorepeats is intriguing. Both codon usage bias and asparagine homorepeats had been the subject of previous studies^14–21^. However, these studies focus either on codon usage bias or the biological role of asparagine homorepeats separately and not with an integrated approach.

Earlier studies on codon usage in *P*. *falciparum* were mainly conducted *in silico*, such as the computing of genome-wide ‘Relative synonymous codon usage’ values as well as describing codon optimality using ‘effective number of codons’ values between highly and lowly expressed genes^15–17, 21^. To complement these *in silico* studies, we used GFP reporter assays to investigate the *in vitro* effect of different codon usages on translation elongation in *Plasmodium falciparum*. Realtime quantitative measurements of GFP signals offer higher sensitivity than traditional protein detection methods that are semi-quantitative. Meanwhile, reliable differentiation of developmental stages can be easily achieved in cytometry analysis to remove this potential confounding effect, conferring additional advantage over the use of luciferase-based reporter assay.

Our results show that the use of GU wobble codons is negatively correlated to protein level. When GU wobble AAT codons were inserted in tandem that is reminiscent of asparagine homorepeats, the insertion also caused destabilization of the transcripts. Furthermore, genome-wide analysis suggests a regulatory role of GU wobble codons in certain categories of genes. We discuss the possible driving forces for the pervasive use of GU wobble codons in the genome and their potential to reframe our understanding of gene regulation in the parasite.

## Results

### Redesigning GFP sequences based on codon usage and codon: anticodon pairing

In this study, we utilized synthetic GFP reporter sequences that were differentially codonized, the sequences were recodonized from GFP_WT_ in the following four ways: an AT-rich sequence with codon usage similar to the genome of *P*. *falciparum*, a GC-rich sequence, a sequence with high-wobble codon content and one with low-wobble codon content.

**Figure 1:**
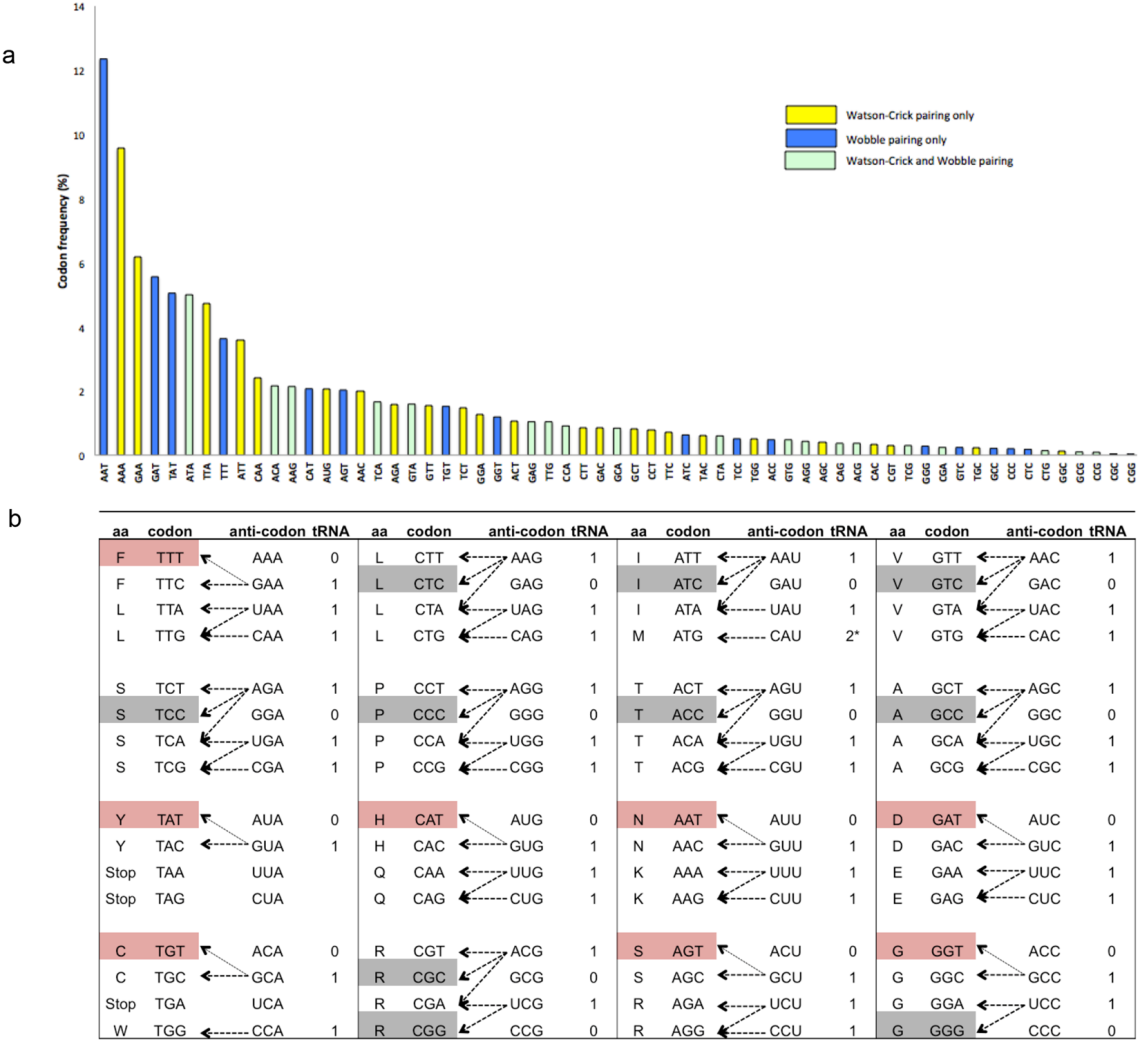
Codon usage and codon-anticodon recognition in *P*. *falciparum*. (**a**) The codon usage frequency in *P*. *falciparum* shows high A+T content in commonly used codons. (**b**) An illustration of all possible codon anticodon pairings predicted by the wobble hypothesis. The number of tRNA gene that contains the anticodon sequence is shown. Red indicates G-U wobble base-pairing codons. Grey indicates non-GU wobble base-pairing codons (i.e. I-C and U-G). *There are two tRNAs with CAU anticodon, one of them is Met-tRNA_i_^Met^ used only for translation initiation.

To aid our design, we first obtained the genome codon usage frequency of *Plasmodium falciparum* (Fig. 1a), and started with codonizing a GFP sequence (GFP_Pf_) that matches the synonymous codons usage frequency of the genome. Unsurprisingly, the genome-wide codon usage frequency reflects the highly skewed genomic A+T content, with the sequence of the ten most frequently used codons containing a total of 90% A+T content, compared to only 10% in the ten least frequently used codons.

For comparison, we also codonized a GFP to the opposite extreme by having a low A+T content. *Leishmania major* has a genomic G+C content of 60%, and is among one of the highest in eukaryotes^22^. Therefore, a codonized GFP (GFP_GC_) according to *L*. *major* synonymous codons usage results in a substantially elevated G+C content (53.9% vs 32.2% in GFP_Pf_).

All *Plasmodium* spp. encode only 45 nuclear tRNA genes, many codons are necessarily decoded by wobble pairings. Before we were able to recodonize GFP into variants that have a high or low wobble codon content, it was necessary to generate a comprehensive view of all possible codon- anticodon pairings in *P*. *falciparum*.

We first aligned all tRNA isoacceptor genes using the universally conserved T33 base and identified the corresponding anticodon that spans position 34–36 (Fig. S1 and Table S1). To verify our identification, we also used tRNAscan-SE to predict the isoacceptors^23^. All predictions matched our alignment results except for PF3D7_1438200. tRNAscan-SE predicted that PF3D7_1438200 to be a Lys-tRNA pseudogene whereas our alignment showed a TCA anticodon sequence, suggesting that it is instead a selenoCys-tRNA reading the TGA codon. The annotation in PlasmoDB supports our alignment result on PF3D7_1438200. However, PF3D7_1252000 is potentially mis-annotated in PlasmoDB as Glu-tRNA since it is predicted as Gln-tRNA in both our alignment and tRNAscan-SE.

We next determined all possible codon: anticodon-pairing following the wobble hypothesis rules^8^. 24 codons are strictly decoded by one isoacceptor through Watson-Crick pairing while 19 codons can be decoded by two isoacceptors, either through Watson-Crick or wobble pairing (Fig. 1b and Table S2). In *P*. *falciparum*, 18 codons encoding 15 different amino acids are decoded necessarily by wobble pairings. Among these, eight of them use GU wobble pairing. Importantly, codons using GU wobble pairing are frequently used in the genome, accounting for 33.4% of the total codon count. Thus, based on our results, we modified the GFP_Pf_ sequence to generate GFP_Wob_ and GFP_Non wob_, which contain a high level and low level of wobble codon respectively (41.6% vs 1.26%).

To minimize the effect due to differential translation initiation, the first 11 nucleotides from the ATG start codon were not altered in all the GFP sequences to retain a strong Kozak context (ACCATGA) as well as to minimize changes on the minimum folding energy surrounding the start codon (hence the 1.26% wobble in GFP_Non wob_). Besides adopting a predefined synonymous codon usage ratio (File S1), all sequences were randomly generated in terms of codon order and were cloned into pARL2 vector.

### GFP abundance correlates with the GU wobble content in a sequence

**Figure 2:**
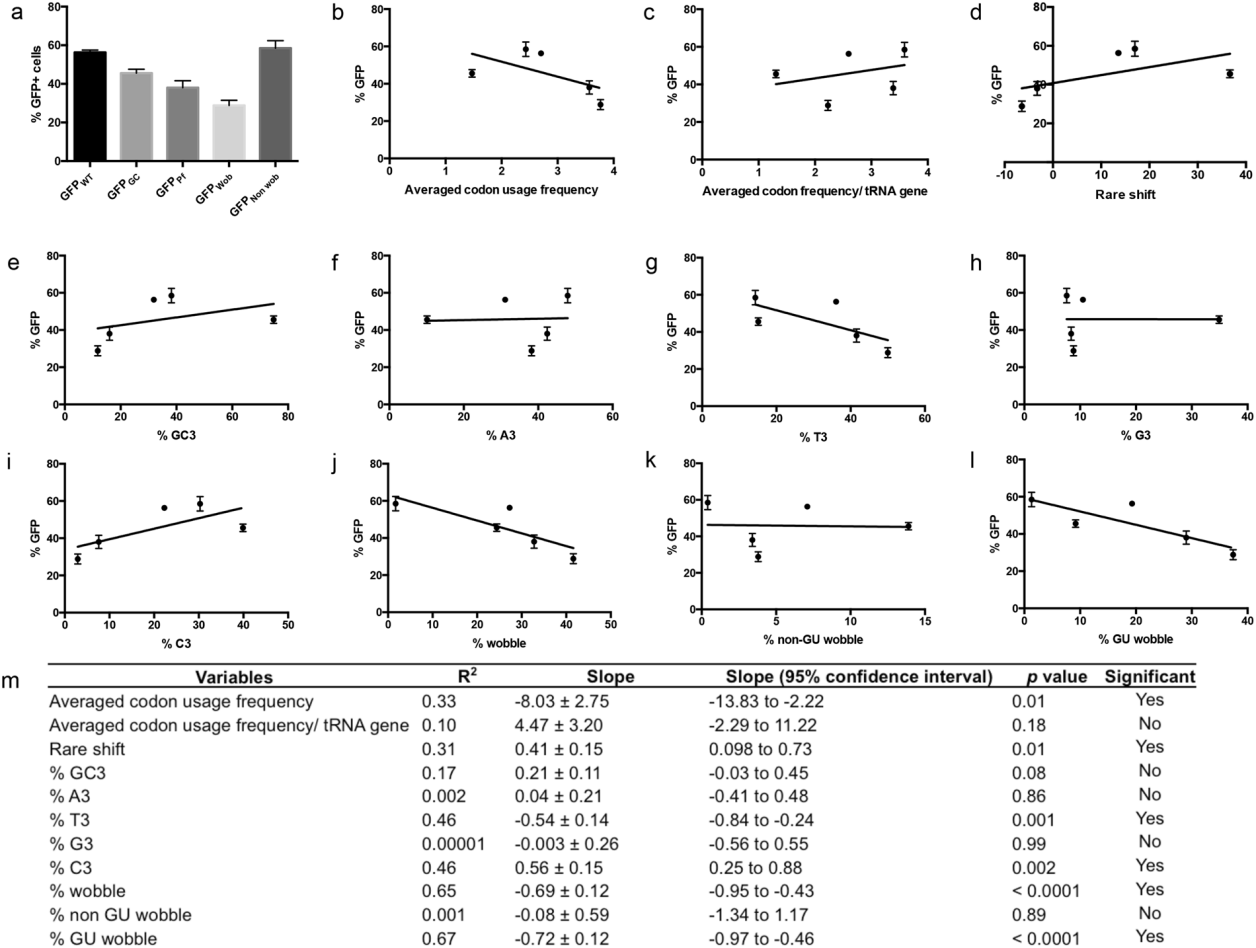
GU wobble pairing reduces GFP signal intensity. (**a**) % GFP+ cells in NF54 culture transfected with different GFP sequence variants. The GFP+ populations are determined in trophozoites population by flow cytometry analysis (n = 4). Major difference can be seen between GFP_Wob_ and GFP_Non wob_. Linear regression analysis between % GFP+ population and (**b**) averaged codon usage frequency, (**c**) averaged codon frequency/tRNA gene, (**d**) rare shift value, (e) % GC3 - codons with G or C in the 3^rd^ position, (**f**) % A3, (**g**) % T3, (**h**) % G3, (**i**)% C3, (**j**) % wobble codon, (**k**) % non-GU wobble codon and (**l**) % GU wobble codon. (**m**) Shows a summary table for all the linear regression analyses. GU wobble content in a sequence is the best predictor of GFP signal intensity as indicated by the R^2^ value. Error bar = S.D.

NF54 parasites were transfected with the GFP sequences and selected by drug to stably maintain the GFP sequences as episomal DNA. We then analyzed GFP signal intensity by cytometry. Since both transcription and translation of the GFP will be affected by the cell cycle progression, signals were only measured in the trophozoites. In each independent experiment, the percentage GFP-high (here termed GFP+) cells and mean fluorescence intensity were highly correlated (R^2^ values between 0.96–0.99, data not shown) and for the purpose of analyses, only the proportion of GFP+ parasites were considered since these values were more consistent between experiments. We observed clear differences in the proportion of GFP+ parasites in the different transfectants (One-way ANOVA *p* < 0.0001, Fig. 2a). Interestingly, GFP_Wob_ and GFP_Non wob_ displayed the greatest difference in the proportion of GFP+ cells, with GFP_Non wob_ showing the highest proportion and GFP_Wob_ showing the lowest (*p* < 0.0001). GFP transcript levels in the transfectants were similar, except for GFP_Pf_ that had reduced GFP transcripts (Fig. S2). Therefore, transcript levels could not correlate and sufficiently explain the signal differences.

Although the GFP sequences were designed to control for G+C and wobble contents, other inter-related variables could also be affected. For example, elevating the G+C content concomitantly uses more rare codons. It also reduces the frequency of wobble codons because many two-box T-ending codons that use GU wobble pairing would be substituted. We therefore established an array of variables to be tested independently for their effects on GFP signals using linear regression analysis.

Average codon usage frequency, average codon frequency/tRNA gene and rare shift value are variables that describe the overall rarity of codons in the sequences. The average codon usage frequency is inversely associated with signal intensity (Fig. 2b). This parameter calculates the averaged genome usage frequency of all codons in the sequence and a higher value corresponds to a higher content of commonly used synonymous codons in the sequence. The inverse relationship between average codon usage frequency and GFP intensity suggest codons more commonly used in the genome are less favorable for efficient translation. It is likely because high frequency use of a codon will increase competition for the corresponding tRNA, making such tRNAs less available during translation. However, there is no significant association when this parameter is corrected for the 19 codons that can be decoded by two isoacceptors (Fig. 2c). This implies tRNA gene copy numbers to the corresponding codons have little influence on translation efficiency, as would have been predicted.

The rare shift values of the sequences also positively associate with signal intensity (Fig. 2d). Rare shift value describes the departure of the observed usage frequency from the expected usage frequency between synonymous codons using the equation:

$$-|\frac{{f}_{o}-{f}_{e}}{{f}_{e}}|,$$

where *f*_o_ is the observed frequency and *f*_e_ is the expected frequency. The expected frequency is computed based on the 81% genomic A+T content (Fig. S3a). In all two-box codon families, the NN(A/T) codon is expected to occur at 81% frequency when encoding the corresponding amino acid. A codon will have a positive rare shift if it is used less frequently than this expected value and vice versa, the rare shift score for the GFP sequences is the summation of the score of each codon. Because rare shift effectively considers the genome-wide selective force acting on each codon regardless of the amino acid composition, it does not necessarily correlate with the codon usage frequency, which is also affected by the genome-wide amino acid composition. However, a weak correlation does exist between codon frequency and rare shift value, with high frequency codons usually used more commonly than expected and therefore having negative rare shift score (Fig. S3b). The association between rare shift and GFP signal also suggests that the replacement of commonly used codons with rare codons improves translation.

Nucleotide compositions in the sequences were also extensively tested for association with GFP signals (Fig. 2e–i). In particular, thymidine content in the wobble positions is inversely correlated while cytosine content in the same position is positively correlated with GFP signal intensity (Fig. 2g,i).

Interestingly, our analyses show that the degree of wobble codon content in the sequence has the most profound effect on GFP signal (Fig. 2j, R^2^ = 0.65, *p* < 0.0001). However, the association is only restricted to the number of GU wobble codons (Fig. 2l, R^2^ = 0.67, *p* < 0.0001), but not with non-GU wobble codons (Fig. 2k, R^2^ = 0.001). None of the other variables shows a better correlation with GFP signals (R^2^ between 0.31 to 0.46). Additionally, removing GFP_Pf_, which showed reduced GFP transcript level, only slightly reduced the R^2^ values (from 0.67 to 0.65).

Notably, six out of the eight GU wobble codons are in the two-box codon families that contain a T-ending wobble codon or a C-ending codon decoded by Watson-Crick pairing. Therefore, an opposite trend between C3 and T3 content reinforces the dependency of GFP signal on the GU wobble content. Moreover, as mentioned above, most GU wobble codons have high usage frequency as well as negative rare shift scores. Therefore, the observed correlations between averaged codon usage frequency, rare shift as well as T3 and C3 content on GFP intensity could be collateral effects secondary to the effect of GU wobble content in the sequence. We conclude that GU wobble content in a sequence is a dominant factor that dictates GFP signal and thus translation efficiency.

### Recodonized sequence enhances translation of endogenous gene

**Figure 3:**
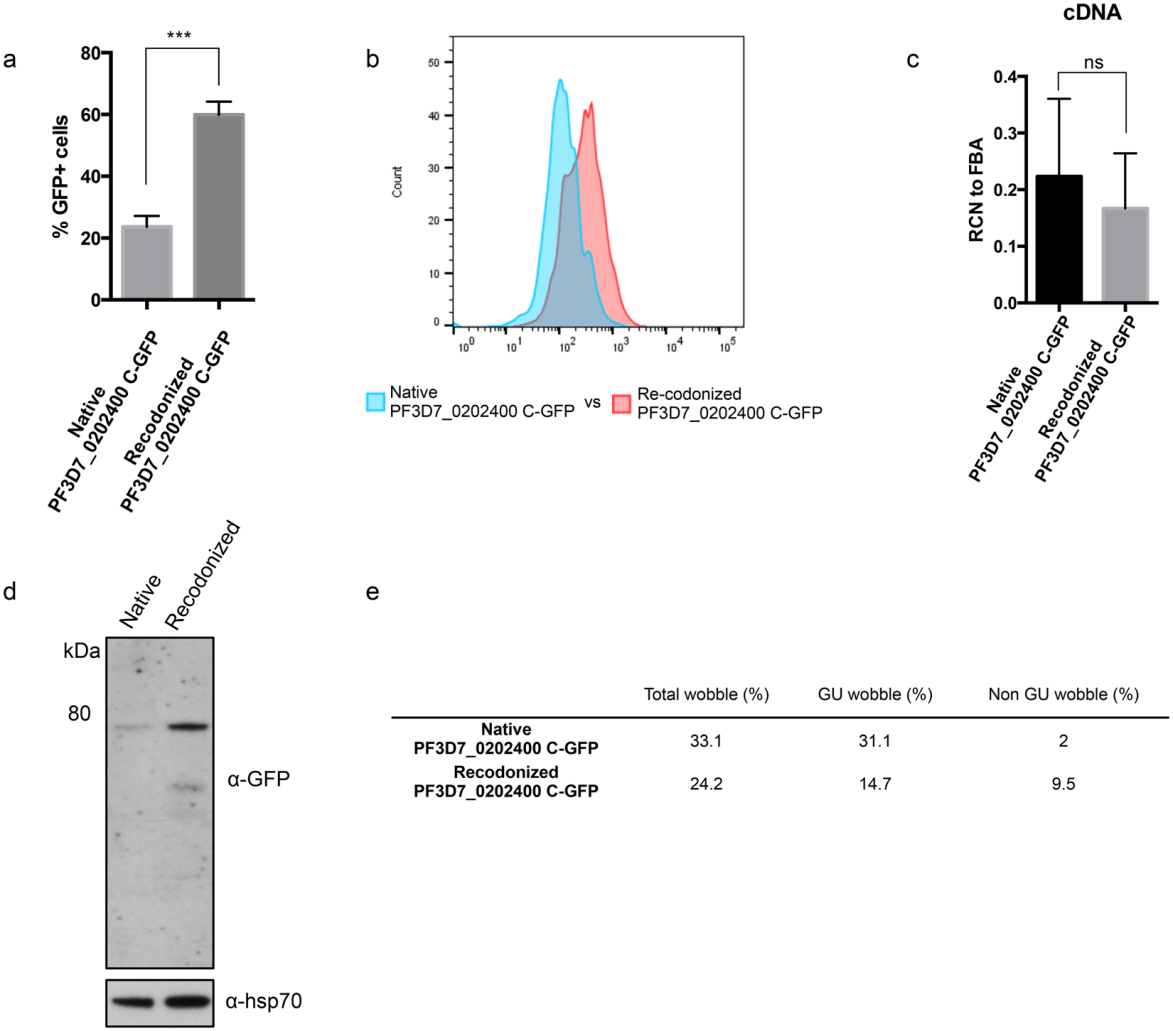
High GU wobble content reduces translation of endogenous sequence. (**a**) % GFP+ cells in NF54 culture transfected with native and recodonized PF3D7_0202400 C-terminus sequence fused to GFP (n = 3, mean ± s.d.). (**b**) The GFP+ populations are determined in trophozoites population by flow cytometry analysis. The entire parasite population generates higher GFP signal when transfected with the recodonized sequence. (**c**) Relative copy number of *gfp* transcript against *fructose biphosphate aldolase* transcript using cDNA from the transfectants (n = 3, mean ± s.d.). (**d**) Western blot analysis on the transfectants with native and recodonized sequence using antibodies against GFP and Pfhsp70 as loading control. There are more GFP fusion proteins detected in transfectants with recodonized sequence. Full-length blots are shown also as supplementary figure. (**e**) Shows the respective wobble codon content in the native and recodonized sequence. Native sequence has more GU wobble codon.

Although GU wobble content reduces GFP signal, its importance may be irrelevant for endogenous genes. Endogenous genes may have evolved adaptations to compensate for the negative effect of GU wobble content. To test this, we overexpressed a GFP_WT_-tagged endogenous gene (PF3D7_0202400, residue 757–1192, which is implicated in pregnancy associated malaria^24^) in NF54 using the native PF3D7_0202400 sequence as well as a recodonized sequence optimized for expression in *E*. *coli*. Resultantly, the total wobble codon content was higher in the native sequence than in the recodonized sequence (33.1% vs 24.2%). However, the high wobble content in the native sequence is exclusively due to GU wobble codon (31.1% vs 14.7%), whereas non-GU wobble codons are actually present in higher number in the recodonized sequence (2% vs 9.5%) (Fig. 3e). Both sequences were cloned into pARL2 with predicted strong translation initiation contexts.

As expected, parasites transfected with the recodonized sequence gave a three-fold greater proportion of GFP+ trophozoites by cytometry (Fig. 3a,b). While detecting no differences in the transcript levels (Fig. 3c), western blot confirmed the difference in protein levels, suggesting the discrepancy at the translational level (Fig. 3d). This result corroborates findings from the GFP reporter assay, confirming that GU wobble codons, but not non-GU wobble codons, reduce translation efficiency.

### Asparagine homorepeat reduces translation due to GU wobble codon identities

**Figure 4:**
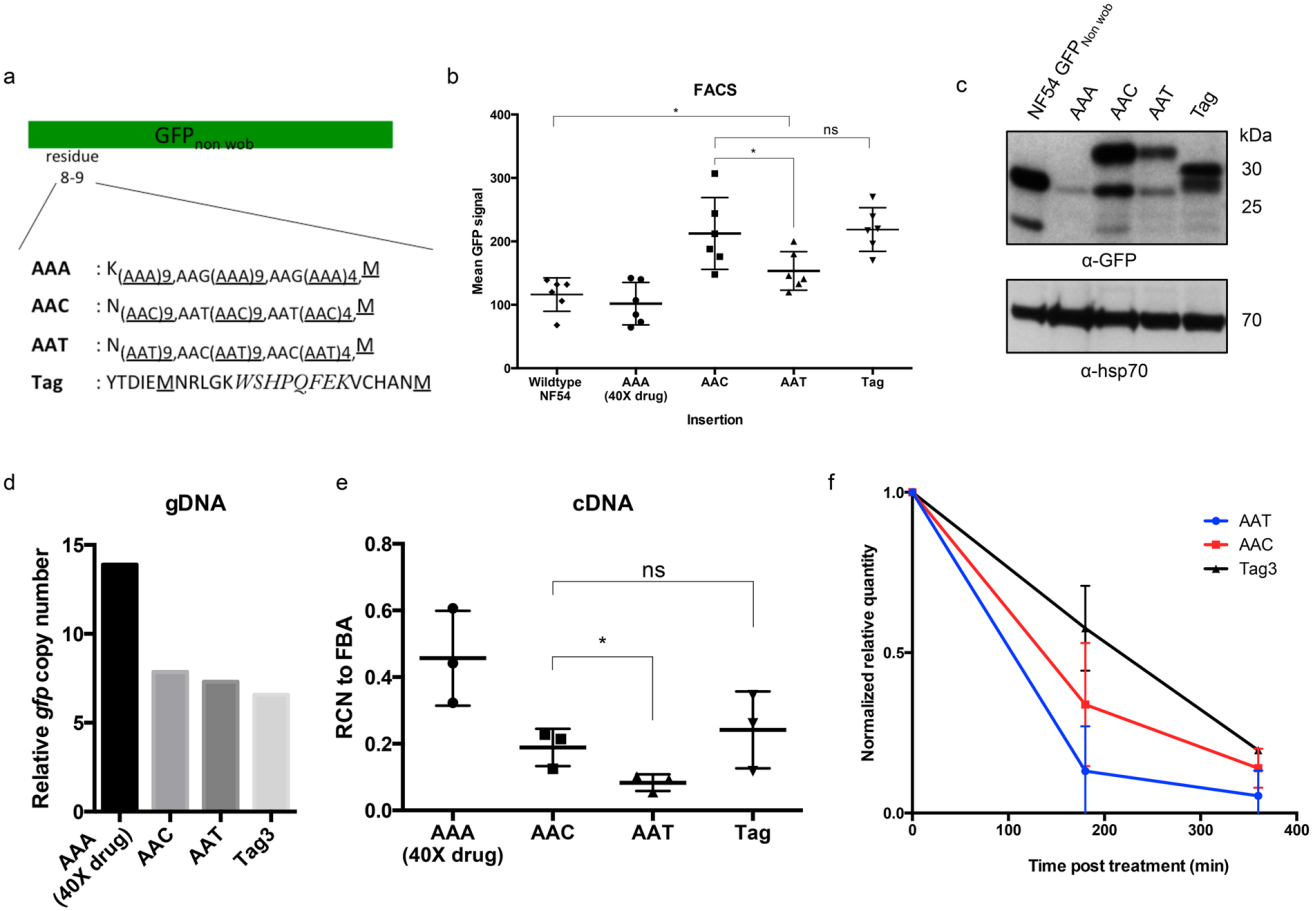
The GU wobble context of asparagine repeats cause reduction of translation. (**a**) Schematic representation of sequences using mostly AAC or AAT codon that mimic simple asparagine repeats found in *P*. *falciparum* genome. AAT insert corresponds to the natural codon frequency used in the genome. AAA serves as negative control, since no protein harbors 24-lysine repeat. Tag insertion is a positive control with totally unbiased codon usage. All sequences were inserted between residue 8 and 9 of GFP_non wob_, avoiding any secondary structure of the GFP. (**b**) Mean GFP signal from NF54 culture transfected with the insertion constructs. AAT insertion causes reduction in GFP signal compared to AAC and Tag insertion (n = 6, mean ± s.d.). (**c**) Western blot analysis on the transfectants with the insertion constructs using antibodies against GFP and Pfhsp70 as loading control. Both AAC and AAT insertion gave the same protein products, but more protein products were detected with AAC insertion. Full-length blots are shown also as supplementary figure. (**d**) qPCR analysis was performed to determine the relative copy number of *gfp* gene against the single copy *fructose biphosphate aldolase* gene. No difference in *gfp* copy number between transfectants with AAC and AAT insertion. (**e**) Relative copy number of *gfp* transcript against *fructose biphosphate aldolase* transcript in cDNA of different transfectants (n = 3, mean ± s.d.). AAT transfectant has lower equilibrium *gfp* transcript level compared to AAC and Tag transfectants. (**f**) Transfectants were treated with 20 ug/ml actinomycin D to block transcription, and the change of the relative *gfp* transcript number against pan 28S rRNA was determined at 180 and 360 minutes after actinomycin D addition. The relative quantity was normalized against time point 0 correspondingly (n = 3, mean ± s.d.). AAT insertion specifically causes more rapid decay.

One of the most frequently used GU wobble codon is the asparagine encoding AAT codon. AAT codon is also the most frequently used codon in the genome, partly due to insertion of asparagine homorepeats in almost 30% of the proteome^25^. While asparagine is also encoded by the AAC codon, the codon usage frequency is hugely different (12.35% vs 2%, Fig. 1a), meaning that 86% of asparagine residues are decoded by AAT. This value is more than expected when adjusted for the genome A+T content (86% vs 81%) or even higher if only the A+T content in the coding region is considered (86% vs 76%). It implies that the increased relative use of AAT codon may have been under a genome-wide positive selection, and that the selection pressure does not act to modulate protein function since synonymous codons will not alter protein function. Since many AAT codons would be compacted in asparagine homorepeats, we reason that the positive selection on AAT codon usage will be most prominently illustrated in these repeats given that they present an extremely biased usage of GU wobble codon. To test this, we constructed two GFP sequences (GFP_AAT_ and GFP_AAC_) with 24 uninterrupted asparagine residues inserted between residue 8 and 9. GFP_AAT_ uses the endogenous AAT/AAC codon ratio (9:1), whereas GFP_AAC_ uses the synonymous codon in every inserted codon compared to GFP_AAT_, resulting in a reverted AAT/AAC ratio (1:9) (Fig. 4a). As a positive control, we also inserted GFP_Tag_ with 24 codons that allow a completely unbiased usage, where no codon will be used twice and all 20 amino acids will be encoded at least once. As a negative control, GFP_AAA_ was inserted with 24 lysine residues since no protein is known to harbor such insertion in the proteome of *P*. *falciparum* (Fig. 4a). We then monitored GFP intensity using cytometry on transfected NF54.

The insertions of these extra codons greatly reduced GFP intensity as well as the proportion of GFP+ parasites (data not shown), even in GFP_Tag_ where codon usage was designed in a completely unbiased fashion. It is likely to be a general negative effect due to the insertion of residues unrelated to the GFP function. Nonetheless, it was still clear that GFP_AAT_ generated significantly weaker GFP signal than GFP_AAC_, with the latter having similar GFP levels to the positive control GFP_Tag_ (Fig. 4b).

Western blot confirmed the difference in translated GFP protein with the insertions (Fig. 4c). Importantly, same bands were detected in GFP_AAT_ and GFP_AAC_, although the bands indicative of full-length insertion were migrating at slightly higher molecular weight than expected. We also detected bands with lower molecular weight in all transfectants that could indicate degradation products. Expectedly, GFP_AAA_ failed to produce GFP despite additional drug pressure (up to 40x) that increased the plasmid copy number (Fig. 4b–d).

Both AAC and AAT codons are decoded by the same tRNA isoacceptor, neither the difference in amino acid availability nor in the charged tRNA pool can explain the differential signal intensity between GFP_AAT_ and GFP_AAC_. Moreover, the extreme amino acid composition has little effect on translation efficiency (both GFP_AAC_ and GFP_Tag_ gave the same GFP levels), our result strongly suggests that the GU wobble codon context underlying AAT codon usage contributes to the reduction in translated GFP product.

Notably, qPCR analysis showed a consistently lower level of GFP transcripts in GFP_AAT_, despite comparable plasmid copy numbers found in all transfectants (excluding GFP_AAA_ which was under additional drug pressure) (Fig. 4d,e). Both transcriptional activity and transcript decay rate can affect equilibrium transcript level, but because all constructs have the same promoter to drive transcription activity, accelerated decay is more likely to account for the reduced GFP transcripts in GFP_AAT_. We performed an assay using actinomycin D to block transcription and then monitored the transcript level after 3 and 6 hours to assess transcript stability and found an increased decay rate of the GFP transcripts in GFP_AAT_ when compared to GFP_Tag_ (Fig. 4f). This suggests a locally high GU wobble content can promote transcript decay and reduces the protein output.

### GU wobble codon usage is reduced in highly expressed genes

**Figure 5:**
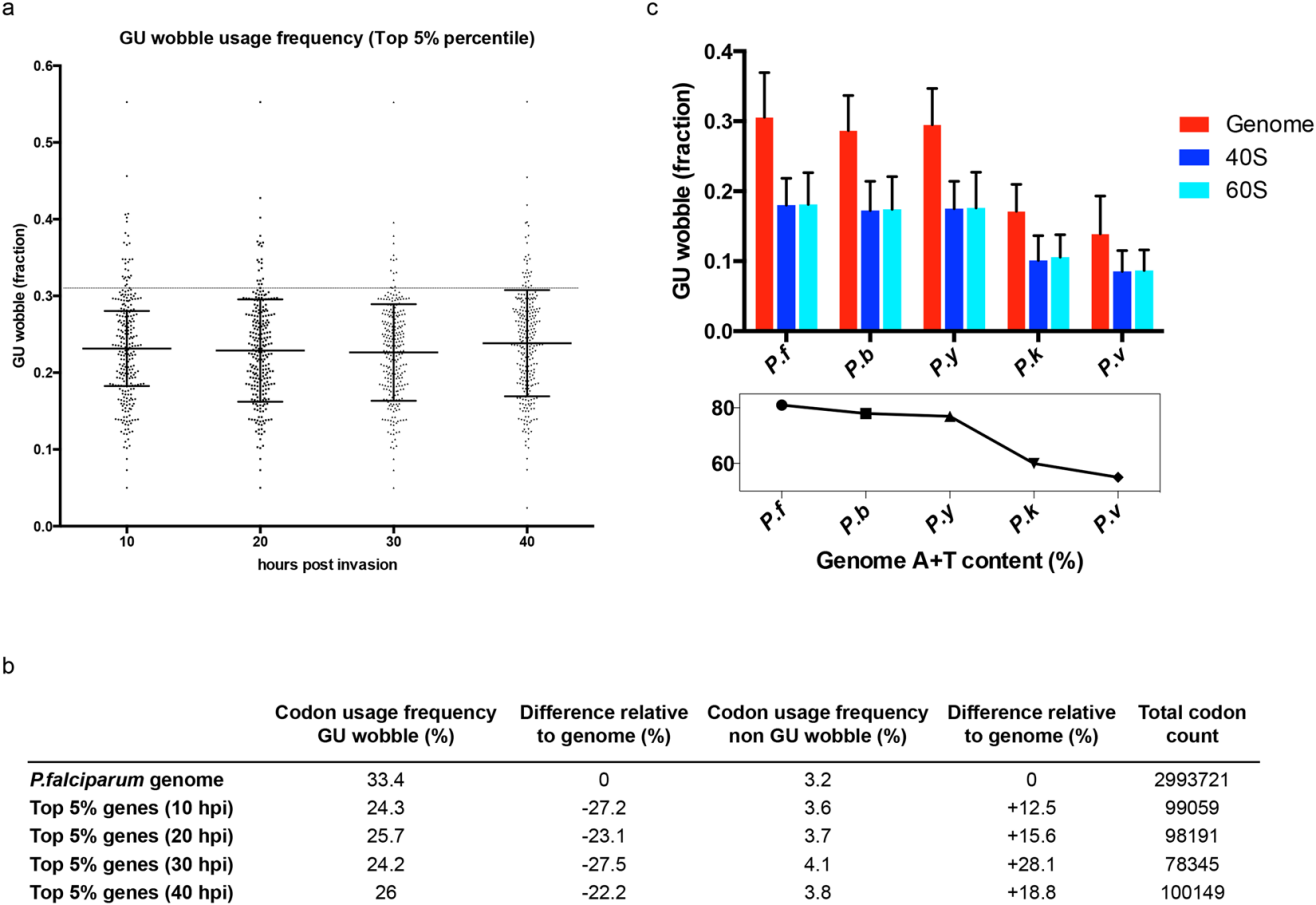
Highly expressed genes have lower GU wobble content. (**a**) The GU wobble content in the top 5% expressed genes at 10, 20, 30 and 40 hours post invasion. Top 5% expressed genes were determined from RNAseq data. The plot shows the mean ± s.d., dotted line represents genome average. (**b**) Comparison between the total wobble codon usage frequencies in top 5% expressed genes and the genome. (**c**) Upper: The GU wobble content in (red) all genes in the genome, (blue) in genes encoding 40S ribosomal proteins and (cyan) 60S ribosomal proteins of five *Plasmodium* spp., *P*.*f*.: *Plasmodium falciparum*, *P*.*b*.: *Plasmodium berghei*, *P*.*y*.: *Plasmodium yoelii*, *P*.*k*.: *Plasmodium knowlesi*, *P*.*v*.: *Plasmodium vivax*. All t-test analyses between genome and 40S/60S in all five species show significant difference between the means. Lower: The A+T content in the different genomes. 40S and 60S ribosomal genes use less GU wobble codon but the extent is dependent on the genomic A+T content.

We postulate that GU wobble content can be a proxy for translational efficiency. If so, highly expressed genes should have lower GU wobble content to maximize translation efficiency. We analyzed the GU wobble content in the top 5% expressed genes during the progression of the asexual stage at 10, 20, 30 and 40 hours post invasion and could clearly see a reduced GU wobble content in these genes when comparing to the genome average (22.6–23.8% vs the genome average of 30.6%) (Fig. 5a). Conversely, non-GU wobble codons are used more frequently when compared to the genome (increase of 12.5% to 28.1%) (Fig. 5b).

We also examined the GU wobble content in genes encoding ribosomal proteins, which are conventionally used as reference genes for describing the optimal codon context in an organism^26^. The GU wobble content in genes encoding 40S and 60S ribosomal subunits are drastically lower than the usage in the genome (mean: 18.0% and 18.1% respectively, vs 30.6% in the genome) (Fig. 5c). This reduction is independent of the amino acid composition because a concomitant increase in the usages of the synonymous codon to all the GU wobble codons represented in the two-box codon families can be seen (i.e. Asn, Asp, Cys, His, Phe, Tyr) (Fig. S4).

Furthermore, the G+C content in these genes is also unlikely to be the primary factor that favors the use of the C-ending synonymous codons over the T-ending GU wobble codons. In the remaining two-box codon families (Glu, Gln, Lys) where both synonymous codons use Watson-Crick pairing, we would expect to see the G-ending synonymous codons preferably used over the A-ending codons if G+C content exerts a similar mutational pressure on codon usage. However this is not the case (Fig. S4), reaffirming that highly expressed genes minimize the use of GU wobble codons to achieve efficient translation.

While other *Plasmodium* spp. may have different codon usage bias, the number of tRNA genes is largely conserved among them. Therefore if GU wobble codons reduce translation efficiency due to the codon-anticodon pairing, it would give a similar effect in other *Plasmodium* spp. Indeed, genes encoding 40S and 60S subunits in *P*. *berghei*, *P*. *yoelii*, *P*. *knowlesi* and *P*. *vivax* also show varying degree of reduction in GU wobble content compared to their respective genomes (Fig. 5c). However, the degree of the reduction is certainly more prominent in the A+T rich genomes of *P*. *falciparum* and *P*. *yoelii* (>10% difference in means). In contrast the reduction in GU wobble content is minimal in *P*. *vivax* (5% difference in means), which only has a genomic A+T content of 55% (Fig. 5c).

Notably, universal decrease in the usage of the synonymous GU wobble codons is true only for asparagine, histidine and tyrosine codon families (Fig. S4). Therefore, while GU wobble codons seem to reduce translation efficiency in *Plasmodium* spp., the magnitudes of the reduction are different across genomes and also different among individual GU wobble codons.

To further validate GU wobble content can affect translation efficiency, we checked if genes with high GU wobble content would have reduced ribosome loading. Efficiently translated transcripts are usually loaded by multiple ribosomes along its length and precipitated in the polysome fractions. Using previously published polysome-profiling data^27^, we compared genes which transcripts were differentially enriched in the polysome fraction and found that genes poorly loaded by ribosomes have higher GU wobble content (Table 1 and Fig. S5). From the dataset, longer genes appeared to be more poorly loaded, which is counter-intuitive since actively translating ribosomes are more likely to be found on longer transcripts^28^. Yet even after controlling for gene length we could still see a difference in GU wobble content between genes highly and poorly enriched in the polysomal fraction (Table 1 and Fig. S5). This suggests a high GU wobble content is also associated with lower ribosome loading, indicative of a reduction in translation efficiency.

**Table 1:** Comparisons of GU wobble frequency between genes with transcripts poorly loaded with ribosomes (Steady state/Polysome >10) and highly loaded with ribosomes (Polysome/Steady State >2).

	Number of genes	Total codon count	Average codon/gene	Total GU wobble usage frequency (%)
All MW considered:				
Steady state/Polysome >10	309	499409	1616	35.8
Polysome/Steady State >2	730	252320	346	29.6
100–300 kDa only:				
Steady state/Polysome >10	115	160576	1396	34.9
Polysome/Steady State >2	43	55526	1291	31.4

Total GU wobble frequency is higher in genes with poorly loaded transcripts.

### High GU wobble content is associated with single-exon genes

**Figure 6:**
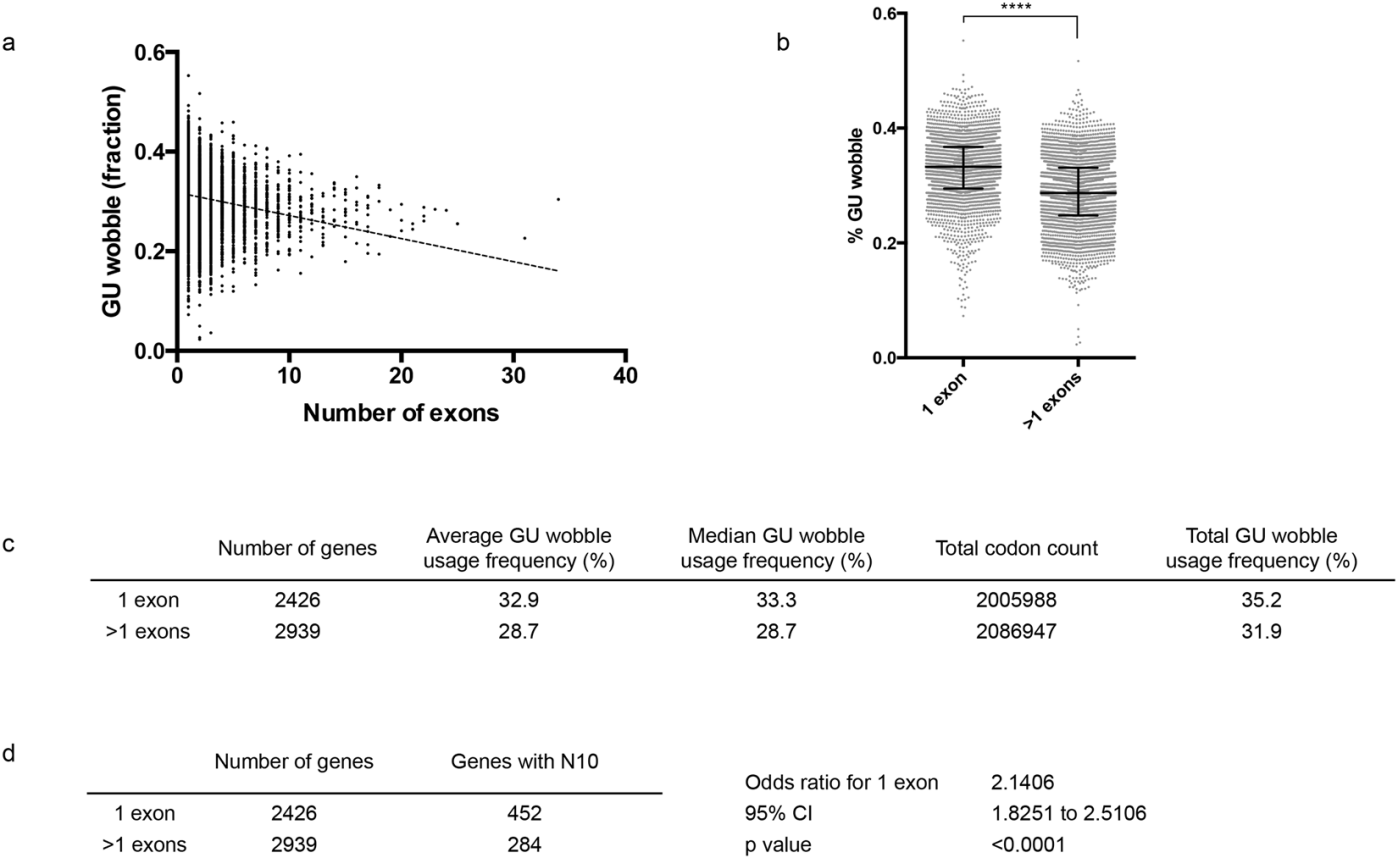
Single-exon genes have higher GU wobble content. (**a**) GU wobble content is correlated to the number of exons, the correlation is demonstrated using all nuclear-encoded genes (Pearson r = −0.2, *p* < 0.0001). (**b**,**c**) Categorical comparison shows higher GU wobble content in single-exon genes compared to multi-exon genes (*t*-*test*: *p* < 0.0001, 95% CI for difference in means = −0.045 to −0.038). (**d**) Single-exon genes are associated with more asparagine repeats (N10) using chi-square test.

When evaluating the GU wobble content in all protein-coding genes, we observed a positive correlation between GU wobble content and protein length (r = 0.41, *p* < 0.0001) (Fig. S6). This is likely because longer proteins more frequently harbor asparagine homorepeats that are largely composed of AAT wobble codon. However, there is also a negative correlation between GU wobble content and number of exons (r = −0.2, *p* < 0.0001) (Fig. 6a). A categorical comparison between single-exon genes and multi-exon genes shows a significantly higher GU wobble content in single-exon genes (Fig. 6b,c). Furthermore, single-exon genes also harbor more asparagine homorepeats (Fig. 6d and Fig. S7). This association suggests generally lower translation efficiency in single-exon genes. Alternatively, the insertion of AAT codons could promote transcript decay (as indicated in our reporter assay), therefore, more rapid transcript turnover maybe underlying many single-exon genes.

### GU wobble content differentiates genes of distinct functions

Lastly, we investigated whether differential GU wobble content usage could have a regulatory role. We performed gene ontology (GO) enrichment analysis on genes that have <20% GU wobble content and found an enrichment of genes involved in immune evasion and host-parasite interaction (Table 2b). For example, the *rif* family genes that encode the RIFIN variable surface antigens (VSA) have a dramatically reduced GU wobble content (mean 19.1%) (Fig. S8). Conversely, analysis on genes with >40% GU wobble content resulted in enrichment of GO terms mostly suggestive of regulatory roles, such as transcription or translation related process. In particular, gene members in the AP2 transcription factor family often have >40% GU wobble codons (Table 2a and Fig. S8).

**Table 2:** Regulatory genes are commonly high in GU wobble content, while genes involved in immune evasion are low in GU wobble content.

GO ID	GO Term	Fold enrichment	Odds ratio	*P*-*value*
**a**.				
GO:2001141	regulation of RNA biosynthetic process	4.93	5.48	2.47E-11
GO:0006355	regulation of transcription, DNA-templated	4.93	5.48	2.47E-11
GO:0051252	regulation of RNA metabolic process	4.8	5.33	4.23E-11
GO:2000112	regulation of cellular macromolecule biosynthetic process	4.29	4.79	1.01E-10
GO:0010556	regulation of macromolecule biosynthetic process	4.29	4.79	1.01E-10
GO:0019219	regulation of nucleobase-containing compound metabolic process	4.26	4.75	1.18E-10
GO:0051171	regulation of nitrogen compound metabolic process	4.26	4.75	1.18E-10
GO:0009889	regulation of biosynthetic process	4.26	4.75	1.18E-10
GO:0031326	regulation of cellular biosynthetic process	4.26	4.75	1.18E-10
GO:0010468	regulation of gene expression	4.06	4.52	3.38E-10
**b**.				
GO:0020033	antigenic variation	8.33	12.64	1.55E-62
GO:0051809	passive evasion of immune response of other organism involved in symbiotic interaction	8.33	12.64	1.55E-62
GO:0051832	avoidance of defenses of other organism involved in symbiotic interaction	8.25	12.51	3.44E-62
GO:0051834	evasion or tolerance of defenses of other organism involved in symbiotic interaction	8.25	12.51	3.44E-62
GO:0051805	evasion or tolerance of immune response of other organism involved in symbiotic interaction	8.25	12.51	3.44E-62
GO:0051707	response to other organism	8.25	12.51	3.44E-62
GO:0043207	response to external biotic stimulus	8.25	12.51	3.44E-62
GO:0052173	response to defenses of other organism involved in symbiotic interaction	8.25	12.51	3.44E-62
GO:0051807	evasion or tolerance of defense response of other organism involved in symbiotic interaction	8.25	12.51	3.44E-62
GO:0052564	response to immune response of other organism involved in symbiotic interaction	8.25	12.51	3.44E-62

(a) The top 10 GO terms enriched in genes with >40% GU wobble content. (b) The top 10 GO terms enriched in genes with <20% GU wobble content.

Although compositional bias in amino acid can give rise to this discrepancy, it is reasonable to suggest that GU wobble use could have been derived as a mechanism to regulate biological processes at a translational level.

## Discussion

In this study we determine that GU wobble pairings are suboptimal for translation in *P*. *falciparum*. These findings are consistent with earlier studies on *P*. *falciparum* codon usage where synonymous C-ending codons in the two–box codon families were described to be the optimal codons^15–17^. Our study also bridges the codon usage bias with the unique expansion of asparagine homorepeats in *P*. *falciparum* proteome.

It had been suggested that asparagine homorepeats are dispensable since deletion of the repeat in the essential gene Rpn6, a proteasome lid subunit, did not generate any phenotypic changes^19^. Moreover, such repeats are only found in the closely related *P*. *reichenowi* but not in other members of the *Plasmodium* genus. Instead, they have been suggested to function as tRNA sponges that slow down the local translation rate and facilitate correct protein folding^18^. It is currently accepted that the limited availability of charged tRNA^Asn^ is crucial in slowing down the decoding of these repeats^20^, but our results point contrary to this. Using a GFP reporter assay, we demonstrated the importance of the codon context that underlies these repeats: it is the high frequency of GU wobble codons encoding these repeats that limits protein output.

Since both GU-wobble and non-wobble codons (AAT and AAC) make use of the same charged tRNA^Asn^ pool, we demonstrate clearly that neither the availability of amino acid nor the charged tRNA^Asn^ pool is a major determinant for protein output. However, our experimental design may have underestimated the importance of amino acid availability in modulating protein output since *in vitro* culture conditions present an excess of amino acids.

While GU wobble content in a gene can limit translation efficiency, it should be emphasized that this cannot in itself be used to predict protein abundance. GU wobble content alone did not explain all the variations seen between the sequences in our GFP assay and other factors would need to be considered. First, G+C content will most likely give rise to more extensive secondary structures in the mRNA transcript. Stable secondary structures can slow down or stall ribosomes during elongation, reducing translation efficiency and appeared to be selected during evolution^29–31^. Second, the codon context and the codon order in the sequence also affect translation^32–34^. Locally clustered synonymous codons can facilitate the reusing of tRNA isoacceptors to improve elongation dynamics. Recent studies have emphasized the importance of di-codon context, which can impart synergetic inhibitory effect on translation dynamics^35^. Third, differential local translation speed along mRNA may enhance correct protein folding^36^. Fourth, recodonization might have inadvertently introduced nucleotide sequence motif that can affect translation efficiency independent of the codon usages. Besides translation elongation, translation initiation is another rate-determining step in eukaryotic protein synthesis and its importance in *P*. *falciparum* is documented^27, 37, 38^. Future studies could shed more light on the relevance and possible hierarchy of all these factors in determining translational output.

The effect of GU wobble codons in reducing translation efficiency has been described before in other organisms. Ribosome profiling in *C*. *elegans* demonstrated higher ribosome occupancy in GU wobble codons than their synonymous codons suggesting they slow down the elongating ribosomes^39^. Similarly, direct measurement of translation rate in *E*. *coli* showed that translation speed was reduced by wobble pairings, although not specifically on GU wobble pairing^40^. Our study suggests this is also the case in the *Plasmodium* genus, yet the extent of the effect of GU wobble codons on the proteome differs significantly among *Plasmodium* species. For example, the GU wobble content is remarkably similar between highly expressed genes and the genome average in *P*. *vivax*. The high G+C content in *P*. *vivax* genome may, in the opposite, favor the use of GU wobble codons to reduce G+C content in the coding region of highly expressed genes, thus minimizing the formation of excessive secondary structures that can hamper translation elongation.

Strikingly, one third of *P*. *falciparum* proteome is encoded by GU wobble codons despite being suboptimal for translation. We propose that codon usage in *P*. *falciparum* is not driven by a selection force to achieve maximal translation efficiency, but that the highly skewed usage of GU wobble codons may function to regulate translation kinetics.

The genome of *P*. *falciparum* has a relatively low number of predicted specific transcription factors and the transcription of many genes is cascaded through cycle progression with little response to environmental perturbation^13, 41^. In this context, GU wobble codons may have been driven to high frequency to act as an important regulatory mechanism.

Human genes subjected to cell cycle fluctuation also commonly adopt non-optimal codon usage. It has been suggested that such a codon context is more permissive to generating a cell-cycle dependent oscillation in protein level, especially when cellular tRNA pools vary^42^. Since the majority of *P*. *falciparum* genes exhibit cell cycle-dependent oscillation, it is unsurprising to see a non-optimal codon context conferred by high GU wobble content in these genes as well. This may have driven high frequency usage of GU wobble in the genome.

Meanwhile, since many regulatory genes have high GU wobble content, it is possible that this allows precise temporal or quantitative regulation in protein levels when responding to environmental changes. Conversely, since immune evasion is an ongoing effort of the parasite, it may not require the same precision of protein regulation and so genes involved in immune evasion are associated with low GU wobble content. Moreover, these genes are also extensively regulated at epigenetic and transcriptional levels, relaxing the need for further translational regulation.

Our finding also suggests that high AAT codon frequency is an important feature that underlies the insertion of asparagine homorepeats in many *P*. *falciparum* proteins. By clustering GU wobble codons, these insertions can reduce protein output by promoting mRNA decay. The intimate relationship between codon optimality and mRNA stability has only recently emerged, with suboptimal codon context destabilizing mRNA^43–47^. In eukaryotes, transcript decay is governed by both normal turnover and mRNA surveillance mechanisms. Nonsense mediated decay (NMD) is a dominant mRNA surveillance pathway that removes defective mRNAs harboring premature stop codon^48^, preventing the synthesis of truncated proteins. Importantly, NMD is only efficiently triggered by exon junction complex, rendering single-exon genes vulnerable to this type of aberration. A high proportion of *P*. *falciparum* genes are without introns and these genes are clearly associated with an elevated GU wobble content and increased frequency of asparagine homorepeats. We posit that single-exon genes may have evolved to adopt suboptimal codon usage to confer rapid transcript decay. In the context of an inefficient NMD pathway, this rapid transcript decay would prevent the accumulation of defective transcripts.

In practical terms, this study provides a reference for codon optimization strategy to overexpress coding sequence in *P*. *falciparum*. Instead of harmonizing the sequence to the endogenous codon usage frequency of the parasite, we recommend that a sequence should be ‘de-wobbled’ to improve protein expression.

In conclusion, we suggest that codon usage bias in *P*. *falciparum* primarily serves a regulatory function, unlike in other organisms where they are optimized for translation efficiency and accuracy. This study furthers our understanding of the evolution of *P*. *falciparum* genome and adds another layer of complexity on its global gene regulation mechanism.

## Methods

### Data source

Codon usage data for *Plasmodium falciparum* 3D7 and *Leishmania major* were taken from Codon Usage database (http://www.kazusa.or.jp/codon/) or calculated from PlasmoDB for all other *Plasmodium* spp. All data source for analyses in this study was obtained from PlasmoDB (http://plasmodb.org) release 28, except for RNA-seq data for the analysis of highly expressed genes, which was generated in our own laboratory (SRA accession: PRJNA374979).

### Cloning

Recodonized GFP sequences were synthesized as G-block fragments (IDT) (File S2). Cloning was performed using Infusion cloning kit (Clontech) in vector. Vector was linearized with Kpn1 and Xma1 (NEB). To generate GFP sequence with codon insertion, primer extension was performed using single-stranded DNA oligos that include the codons for the first 8 residues of the GFP and the corresponding codon insertion. Cloning was performed using Infusion cloning (Clontech) with Kpn1 and Xma1 linearized pARL2-GFP_non wob_ vector. All clonings were verified with sequencing. All primers used are included (File S6).

### Parasite culture, transfection and transcription blocking assay

Standard method described previously was used to maintain *Plasmodium falciparum* NF54 strain^49^. Cultures were kept at 3% hematocrit and were regularly stage-synchronized with 5% sorbitol.

Parasites were transfected with the red blood cell (RBC) pre-loading protocol^50^. Briefly, 150 µg of plasmid DNA were mixed with 400 µl of freshly washed RBCs and resuspended in cytomix to a total volume of 800 µl. This mixture was transferred and electrophorated in a 0.2 cm cuvette (Bio-Rad) using a Gene Pusler Electroporation System (Bio-Rad) on an exponential decay program set to 0.31 kV and 950 μF. Schizont-infected RBCS were co-cultured with the transfected RBCs. Pyrimethamine (50 nM; Sigma) was added to the culture until parasitemia exceeded 5%, usually after 24 hours, to select for resistant parasite.

For transcription blocking, 20 ug/ml actinomycin D was added to the culture. The cultures were collected for sampling after 3 and 6 hours of incubation.

### Cytometry analysis

100 µl of synchronized cultures were stained with 5 µl Hoechst33342 (200 ug/ml) and 0.5 µl of dihydroethidium (1 mg/ml) for 1 hr at room temperature. 30 µl of stained cells were added to 150 µl PBS in 96-well plates and analysed by BD FACSVerse flow cytometer. FITC levels of trophozoite-infected cells were measured after sequential gating as described elsewhere^51^, with only singlet events being measured. GFP+ cells were defined as all events that generated FITC signals above the untransfected NF54 parasites.

### Genomic DNA extraction

10x volume of 0.1% saponin in PBS was used to lyse 100 µl of infected RBC. DNeasy Blood and Tissue kit (Qiagen) was used to extract DNA from the resultant parasite pellet.

### RNA extraction and cDNA synthesis

Parasites were synchronized for three consecutive cycles before RNA extraction. 100 µl of infected RBC was lysed in 1 ml Trizol reagent (Ambion), total RNA was extracted according to manufacturer’s recommendations. 500 ng total RNA was taken to synthesize cDNA using iscript reverse transcriptase kit (Bio-Rad).

### Quantitative PCR analysis

qPCR was performed using iQ SYBR Green Supermix (Bio-Rad). All qPCR protocols were run for 40 cycles of 95 °C for 10 s and at 60 °C for 1 min, fluorescence signals were read during the elongation step. 2^−ΔCt^ method was used to analyze the results using fructose biphosphate aldolase or 28S rRNA (with cDNA diluted 1:500) for normalization. All primers used are included (File S6).

### RNA sequencing

RNA samples were collected at 10, 20, 30 and 40 hours post invasion. Bio-analyzer (Agilent) was used for quantification and quality assessment of the samples. Sequence library was constructed with 2 ug total RNA using TruSeq Stranded mRNA library Prep kit (Illumina) and sequenced on the Illumina Hiseq 2000 platform. 7.26 to 9.8 million 2 × 100 bp paired-end reads were obtained. Uniquely mapped reads, including duplicated reads, were aligned to the 3D7 reference genome (v9.0) using STAR^52^. Read counting and RPKM values for all genes were generated using HTseq. The RPKM values for all genes are included (File S3).

### SDS-polyacrylamide gel electrophoresis and immunoblotting

Infected RBCs (>1 × 10^7^) were lysed using 0.1% saponin in PBS and washed three times with PBS. Cell pellets were dissolved in NuPAGE LDS loading buffer (Invitrogen) supplemented with NuPAGE sample-reducing agent (Invitrogen) at 1:15 v/v ratio. NuPAGE Novex 10% Bis-Tris gels were used to resolve the cell lysates using electrophoresis. MOPS buffer (Invitrogen) was used for the electrophoresis. After running the gel, resolved proteins were transferred from the gel to nitrocellulose membrane in transfer buffer (25 mM Tris, 192 mM glycine, 20% methanol, 0.025% SDS). The membrane was incubated in 1% western blocking reagent (Roche) in TBS overnight at 4 °C. Afterwards, mouse anti-GFP (1:500, Roche #11814460001) and rabbit anti-PfHsp70 (1:10,000, BioSite SPC-186C/D) were diluted in 0.5% blocking buffer in TBS. The membrane was incubated for 1 hour at room temperature and subsequnetly washed thrice in TBST. The membrane was then incubated for 45 minutes at room temperature with HRP-conjugated secondary antibodies (1:10,000, GE healthcare). For detection, ECL prime western blotting reagent was used and developed on ECL hyperfilm (GE healthcare).

### Polysome enrichment analysis

Polysome enrichment analysis was performed using data deposited in PlasmoDB^27^. To perform the analysis, we took all genes that have RPKM fold change between steady/polysome fraction >10 (to represent genes of very low transcript polysome loading) and genes that have RPKM difference between steady/polysome fraction <0.5 (or polysome fraction/steady fraction >2, to represent genes with relatively high loading) at either 18 or 36 hours post invasion. A *p*-value < 1e-10 in fisher’s exact test was set as the cutoff for fold change. We used database version 28 and did not include data on timepoint 0 because translation is relatively less active at this timepoint. Extended results for the analysis are included (File S4).

### Gene ontology enrichment analysis

GO term analysis was performed with the in-built tool in PlasmoDB. Ontology was searched based on ‘biological process’ with a *p*-value cutoff of 0.05. Extended results for the analysis are included (File S5).

## Electronic supplementary material

Supplementary info

Supplementary info

Supplementary info

Supplementary info

Supplementary info

Supplementary info

Supplementary info
