# Supplementary material for: Frequent GU wobble pairings reduce translation efficiency in Plasmodium falciparum
Source: Sci Rep. 2017 Apr 7;7:723. doi: 10.1038/s41598-017-00801-9 (PMC5429705; doi:10.1038/s41598-017-00801-9)
Supplement: Supplementary file 1 — Supplementary info [file 41598_2017_801_MOESM1_ESM.pdf]

Supplementary Information for:

Frequent GU wobble pairings reduce translation efficiency in Plasmodium falciparum

Sherwin Chan, Jun-Hong Ch'ng, Mats Wahlgren, Jessada Thutkawkorapin

Supplementary Figure Legends

Figure S1. Sequence alignment of all nuclear tRNAs. The alignment was performed with ClustalW2. The conserved T33 is marked with *, anticodons are highlighted in the red box. The T-loops are shown in blue box.

Table S1. A list of all nuclear-encoded tRNA genes is shown.

Table S2. A list of all possible codon-anticodon pairs predicted according to the wobble hypothesis. Adenosine in the wobble position of the anticodon is commonly deaminated to inosine (I), which can pair with a U, A or C base. WC: Watson-Crick pairing with cognate tRNA, Wob: wobble pairing. Secondary wobble pairing denotes codons that have the possibility to pair with another tRNA secondary to the cognate tRNA through wobble pairing.

Figure S2. GFP transcript level in transfectants with different recodonized GFP constructs. qPCR analysis was performed to determine the relative copy number of *gfp* transcript against the *fructose biphosphate aldolase* (n=4). The transcript level of GFP_{pf} is significantly lower when compared to other transfectants.

Figure S3. (a) The rare shift values of all codons, negative value means the codons are used more than expected after adjusting to genome A+T content, and vice versa. (b) Correlation between rare shift value and codon usage frequency in the genome (Pearson r = -0.32, p = 0.01).

Figure S4. The ratios of synonymous codon frequency of all two-box codon families are shown. In (red) the genome, (blue) in genes encoding 40S ribosomal proteins and (cyan) 60S ribosomal proteins of five *Plasmodium* spp., *P.f.*: *Plasmodium falciparum*, *P.b.*: *Plasmodium berghei*, *P.y.*: *Plasmodium yoelii*, *P.k.*: *Plasmodium knowlesi*, *P.v.*: *Plasmodium vivax*. Panels above the dotted line correspond to two-box codon families with T or C-ending codon, panels below the dotted line correspond to two-box codon families with A or G-ending codon.

Figure S5. GU wobble content in genes with low and high ribosome loading. Left panel shows the GU wobble content in genes with transcripts poorly loaded with ribosome (Steady state/ Polysome >10) and highly loaded with ribosome (Polysome/ Steady State >2). Right panel shows genes stratified base on the molecular weight of the gene products (100kDa-300kDa).

Figure S6. GU wobble content is correlated with protein length. Higher GU wobble content is associated with longer protein (Pearson r = 0.41, p <0.0001).

Figure S7. Asparagine homorepeats are more likely to be found in single-exon genes. Percentage of genes that contain increasing number of uninterrupted tandem asparagine codons (10-30), genes are categorized into 1-exon (n=2426) or >1-exon genes (n=2939).

Figure S8. GU wobble contents in different gene families. Members of the AP2 transcription factor family have high GU wobble content. Whereas, *rif* genes are usually low in GU wobble content.

Figure S9. Supplementary full-length blot images. (a) For figure 3C panels; (b) for figure 4C panels.

FigS1

A

```
PF3D7_0203500        ------GGTTCTGTAGTGTAG-TGGTTAGCACTGCAGACTCTGA--------------CT 39
PF3D7_1252000        ------GGTTTCGTAGTGTAG-TGGTTAGCACTGAGGACTTTGA--------------AT 39
PF3D7_0706900        ------GTCCAAATCGTCTAG-TGGTTAGGACTCCACGCTGTGG--------------AC 39
PF3D7_0411600        ------TCCCACGTGGTCTAG-TGGCTAGGATATTCGGCTCTCA--------------CC 39
PF3D7_0527700        ------TCCCATGTAGTCTAGGCGGTTAGGATATTCGGCTTTCA--------------CC 40
PF3D7_0714700        ------TCCGAGATAGTATAG-TGGCAAGTATTTCCGCCTGTCA--------------CG 39
PF3D7_0730600        ------GCGGGCATGGTCTAG-TGGCTATGACGCCTGCCTTACA--------------CG 39
PF3D7_1251900        ------GCGGGCATGGTCTAG-TGGCTATGACGCCTGCCTAACA--------------CG 39
PF3D7_0312600        ------GCGAGCATGGTCTAG-TGGCTATGACGTTCGCCTCACA--------------CG 39
PF3D7_1103200        -----GGCGTCAATAGTCTAA-CGGCCATGATACCTGCCTTCCA--------------AG 40
PF3D7_1370200        ------GCATCTGTGGTCTAG-TGGT-AGAATACTTCGTTGCCA--------------TC 38
PF3D7_1216800        ------GGCTACTTGATCTAG-TGGT-ATGATTCTTGCTTCGGG--------------TG 38
PF3D7_1418400        ------GGCTACTTAGTCTAG-TGGT-ATGATTCTCTCTTAGGG--------------TG 38
PF3D7_1339200        ------GGCTACTTAGTCTAG-TGGT-ATGATTCTCGCTTTGGG--------------TG 38
PF3D7_0411500        ------GGGCAGGTGGTGTAG-TGGT-ATCACGCTTGATTTGCA--------------TT 38
PF3D7_0620800        ------GGGCTAGTAGTGTAG-TGGT-ATCACGTTCGCTTCGCA--------------TG 38
PF3D7_0707000        ------GCTGCCTTAGCTCAGTCGGT-AGAGCGCCAGACTCTTA--------------AT 39
PF3D7_0707100        ------GCCTCTTTAGCTCAGTTGGT-AGAGCGCCAGACTTTTA--------------AT 39
PF3D7_0730700        ------GCCACCTTAGCACAGT-GGT-AGTGCGTTGGTCTTGTA--------------AA 38
PF3D7_1355400        ------GCCATCTTAGCACAGTTGGT-AATGCGTTTGATTCGTA--------------AT 39
PF3D7_0410200        ------GATCCATTAGCTCAGT-GGTTAGAGCGTCGGTCTTATG--------------TA 39
PF3D7_1438300        ------GGGTAATTGGCGCAGTTGGTTAGCGCGCGGGTCTCATA--------------AT 40
PF3D7_0403000        ------GGTTCCGTAGCTCAGTTGGTTAGAGCGTGCGGCTGTTA--------------AC 40
PF3D7_1339100        ------AGCAGCGTAGCTCAG--AGGAAGAGTGGGGGGCTCATA--------------AC 38
PF3D7_0312700        ------GGTCCTATAGCTCAGTTGGTTAGAGCGTACGGCTAATA--------------AC 40
PF3D7_0514400        ------GCCGTGATAGCTCAGTT-GGGAGAGCGTCAGACTGAAG--------------AT 39
PF3D7_0702800        ------CCGATGATAGCTCAGTT-GGTAGAGCGGCAGACTGTAGTTGAAATGGT---TAT 50
PF3D7_0706800        ------GCCTGCATAGCTTAGTT-GGTAGAGCATCCGCCTAGTA--------------AG 39
PF3D7_1369900        ------GGGCCTATGGCGCAAC--GGTAGCGCGTCTGACTCCAG--------------AT 38
PF3D7_1370100        ------GGGCGTGTAGCTCAGC--GGTAGAGCAGCTGACTGCAG--------------AT 38
PF3D7_1369800        ------GGGCCGGTAGTTCAGTTGGATAGAATGCCCGACTACGG--------------AT 40
PF3D7_0702700        ------GGGCGACTAGCTCAAGT-GGTAGAGCGCTCGCTTAGCA--------------TG 39
PF3D7_1341000        ------GCCTCTGTGGCGCAATTGGATAGCGCGTTGGACTTCTA--------------AT 40
PF3D7_1370000        ------GCACCAGTGGCGTAATTGGATAGCGCAATGCCTTCCTA--------------AG 40
PF3D7_0529600        ------GACGGCGTAGCCTAAT-GGATAAGGCGTCGGTCTTCGG--------------AA 39
PF3D7_0621600        ------GACAGTTTGCCCGAGTGGT-TAAGGGGTTGGACTTGAAATCCAATGA----GCT 49
PF3D7_1337600        ------GACAGTTTGTCCGAGTGGT-TAAGGAGGTTGACTCGAAATCAACTGG----GCT 49
PF3D7_0410100        ------GACAGTGTGTCCGAGTGGT-TAAGGAGTCAGACTAGAAATCTGGTAG----GCT 49
PF3D7_0714900        ------GATAACGTGCCCGAGTGGT-TAAGGGGTTGGACTGCTAATCCAATGG----GTT 49
PF3D7_1438200        CAATGCACCGATGAGTTAGCATGGT-TGCTAAGTATGACTTCAAATCATTTGGCGTAGTT 59
PF3D7_0510600        ------GTCAGGATGGCCGAGTGGTCTAAGGCGCAGCGTTTAGG---CCGCTG----TCC 47
PF3D7_0714800        ------GACAGAATGGCCGAGTGGTCTAAGGCGCAACGTTAAGG---CCGTTG----TCC 47
PF3D7_0620900        ------GACAGAATGGCCGAGCGGTCTAAGGCGCTACAGTCAGA---TTGTAG----TCT 47
PF3D7_0527800        ------GCACGGGTGCCCGAGTGGT-TAAGGGGGTGGACTTAAGATCCTCTGG----TCA 49
PF3D7_1103300        ------GCACGGATGGCTGAGTGGTCTAAAGCGTCAGACTCAAGATCTGATGA----ACG 50
                                                            *
```

FigS1 (cont.)

```
PF3D7_0203500      CTGCAAA-CCTGGGTTCAAATCCCAGCAGAAC-CT--------------- 72
PF3D7_1252000      CCTCCAA-CCCGGGTTCGAGTCCCGGCGAGAC-CT--------------- 72
PF3D7_0706900      GTGGCAA-CGTAGGTTCGAATCCTGCTTTGGA-CA--------------- 72
PF3D7_0411600      CGAAAGG-CCCGGGTTCAATTCCCGGCGTGGG-AA--------------- 72
PF3D7_0527700      CGAACGA-CCCGGGTTCGAGTCCCGGCGTGGG-AA--------------- 73
PF3D7_0714700      CGGAAGA-CCCGGGTTCAATTCCCGGTCTCGG-AG--------------- 72
PF3D7_0730600      CAGGAGATCCCGAGTTCGATCCTCGGTGCCCG-TA--------------- 73
PF3D7_1251900      CAGGAGATCCCGAGTTCGATCCTCGGTGCCCG-TA--------------- 73
PF3D7_0312600      CGAAAGATCCCGAGTTCGATCCTCGGTGCTCG-TATTAA----------- 77
PF3D7_1103200      CAGGTGA-CCCGGGTTCGACTCCCGGTTGACG-CA--------------- 73
PF3D7_1370200      GAAGTGA-CCCGGGTTCGATTCCCGGCAGATG-CA--------------- 71
PF3D7_1216800      CAAGACGTCCCGGGTTCGATTCCCGGAGTAGC-CC--------------- 72
PF3D7_1418400      GGAGAGGTCCCGGGTTCGATTCCCGGAGTAGC-CC--------------- 72
PF3D7_1339200      CGAGAGGTCCCGGGTTCAATTCCCGGAGTAGC-CC--------------- 72
PF3D7_0411500      CAAGAGGTCCGGGGTTCAATTCCCCGTCTGTC-CA--------------- 72
PF3D7_0620800      CGAAAGTTCCCGGGTTCGATTCCCGGCTGGTC-CA--------------- 72
PF3D7_0707000      CTGGTGGTCAGGGGTTCGAGCCCCCTAGGCAG-CT--------------- 73
PF3D7_0707100      CTGGTGGTCAGGGGTTCGAGTCCCCTAGGAGG-CT--------------- 73
PF3D7_0730700      CCAAAGGTCGGGAGTTCGATCCTCCCAGGTGG-CT--------------- 72
PF3D7_1355400      CAAAAGATCGTGGGTTCGACTCCCACAGGTGG-CT--------------- 73
PF3D7_0410200      CCGAAGGTCGTGGGTTCGAAACCCACATGGAT-CA--------------- 73
PF3D7_1438300      CCCGAGGTCGTGAGTTCGATCCTCACATTACC-CA--------------- 74
PF3D7_0403000      CGCAAGGTCGTTGGTTCGATCCCAGCCGGTAC-CG--------------- 74
PF3D7_1339100      CCCCAGGACCGTGGATCGAAACCACGCGCTGC-TA--------------- 72
PF3D7_0312700      CGTAAGGTCGGCGGTTCGAGACCGCCTGGGAC-CAAA------------- 76
PF3D7_0514400      CTGAAGGTCCCTGGTTCGATCCCTGGTCACGG-CA--------------- 73
PF3D7_0702800      CTGTTGGTCACCGGTTCGATTCCGGTTCATCG-GA--------------- 84
PF3D7_0706800      CGGAAGGTCATCGGTTCGACTCCGGTTGTAGG-CT--------------- 73
PF3D7_1369900      CAGAAGGCTGGGGGTTCGAATCCCTCTGGGCT-CA--------------- 72
PF3D7_1370100      CAGCGGGTCCATGGTTCAAATCCGTGCGCGCC-CT--------------- 72
PF3D7_1369800      CGGGAGGTCGTGGGTTCGACTCCTGCCCGGCC-TA--------------- 74
PF3D7_0702700      CGAGAGGTACGGGGATCGATACCCCGGTCGTC-CA--------------- 73
PF3D7_1341000      CCAAAGGCTGCGGGTTCGAATCCCGCCAGGGG-TA--------------- 74
PF3D7_1370000      GCAAAGGTTATGGGTTCGAGTCCCATCTGGTG-TA--------------- 74
PF3D7_0529600      CCGAAGATTGCGGGTTCGAGTCCCGTCGTCGT-TA--------------- 73
PF3D7_0621600      TTGCT-TGCGCAGGTTCGAATCCTGCAGCTGT-CG--------------- 82
PF3D7_1337600      CTGCC-CGCACAGGTTCAAATCCTGTAGCTGT-CG--------------- 82
PF3D7_0410100      TTGCC-TGCGCAGGTTCGAATCCTGCCGCTGT-CG--------------- 82
PF3D7_0714900      CTGCC-CGCGCAGGTTCGATTCCTGCCGTTGT-CG--------------- 82
PF3D7_1438200      TTTCTGCGCAGAGGTTCGATTCCTCCTTCGGTGCGACGAGCACCAA---- 105
PF3D7_0510600      GTA-AGGGCGTGGGTTCGAACCCCACTCCTGA-CA--------------- 80
PF3D7_0714800      GAA-AGGGCGTGGGTTCGAACCCCACTTCTGT-CA--------------- 80
PF3D7_0620900      GTATAAGGCGTGGGTTCGAATCCCGCTTCTGT-CA--------------- 81
PF3D7_0527800      CTAGACCGCGTGGGTTCGAACCCCACCTCGTG-CA--------------- 83
PF3D7_1103300      TAAGTTCGCATGGGTTCGAACCCCATTTCGTG-CATATTTAAAAAGGCCA 99
                                * ** *  *
```

TableS1

Gene ID	Anti-codon 5'-3'	Isotype
PF3D7_0702700	AGC	Ala
PF3D7_0411500	UGC	Ala
PF3D7_0620800	CGC	Ala
PF3D7_1370100	GCA	Cys
PF3D7_0714700	GUC	Asp
PF3D7_0527700	UUC	Glu
PF3D7_0411600	CUC	Glu
PF3D7_0514400	GAA	Phe
PF3D7_1370200	GCC	Gly
PF3D7_1103200	UCC	Gly
PF3D7_0706900	GUG	His
PF3D7_0312700	AAU	Ile
PF3D7_0410200	UAU	Ile
PF3D7_0707000	CUU	Lys
PF3D7_0707100	UUU	Lys
PF3D7_0527800	UAA	Leu
PF3D7_1103300	CAA	Leu
PF3D7_0714800	AAG	Leu
PF3D7_0510600	UAG	Leu
PF3D7_0620900	CAG	Leu
PF3D7_1438300	CAU	Met
PF3D7_0403000	GUU	Asn
PF3D7_1418400	AGG	Pro
PF3D7_1339200	UGG	Pro
PF3D7_1216800	CGG	Pro
PF3D7_1252000	UUG	Gln
PF3D7_0203500	CUG	Gln
PF3D7_1341000	UCU	Arg
PF3D7_1370000	CCU	Arg
PF3D7_1369800	ACG	Arg
PF3D7_0529600	UCG	Arg
PF3D7_0714900	GCU	Ser
PF3D7_0410100	AGA	Ser
PF3D7_0621600	UGA	Ser
PF3D7_1337600	CGA	Ser
PF3D7_0706800	AGU	Thr
PF3D7_0730700	UGU	Thr
PF3D7_1355400	CGU	Thr
PF3D7_1251900	AAC	Val
PF3D7_0730600	UAC	Val
PF3D7_0312600	CAC	Val
PF3D7_1369900	CCA	Trp
PF3D7_0702800	GUA	Tyr
PF3D7_1339100	CAU	iMet
PF3D7_1438200	UCA	SelCys

TableS2

Amino acid	Codon 5'-3'	Anti-codon 5'-3'	Type of pairing	Binding at third position	Secondary wobble pairing
A	GCT	AGC	WC	I:U	
A	GCC	AGC	Wob	I:C	
A	GCA	UGC	WC	U:A	I:A
A	GCG	CGC	WC	C:G	U:G
C	TGT	GCA	Wob	G:U	
C	TGC	GCA	WC	G:C	
D	GAT	GUC	Wob	G:U	
D	GAC	GUC	WC	G:C	
E	GAA	UUC	WC	U:A	
E	GAG	CUC	WC	C:G	U:G
F	TTT	GAA	Wob	G:U	
F	TTC	GAA	WC	G:C	
G	GGT	GCC	Wob	G:U	
G	GGC	GCC	WC	G:C	
G	GGA	UCC	WC	U:A	
G	GGG	UCC	Wob	U:G	
H	CAT	GUG	Wob	G:U	
H	CAC	GUG	WC	G:C	
I	ATT	AAU	WC	I:U	
I	ATC	AAU	Wob	I:C	
I	ATA	UAU	WC	U:A	I:A
K	AAA	UUU	WC	U:A	
K	AAG	CUU	WC	C:G	U:G
L	TTA	UAA	WC	U:A	
L	TTG	CAA	WC	C:G	U:G
L	CTT	AAG	WC	I:U	
L	CTC	AAG	Wob	I:C	
L	CTA	UAG	WC	U:A	I:A
L	CTG	CAG	WC	C:G	U:G
M	ATG	CAU	WC	C:G	
N	AAT	GUU	Wob	G.U	
N	AAC	GUU	WC	G:C	
P	CCT	AGG	WC	I:U	
P	CCC	AGG	Wob	I:C	
P	CCA	UGG	WC	U:A	I.A
P	CCG	CGG	WC	C:G	U:G
Q	CAA	UUG	WC	U:A	
Q	CAG	CUG	WC	C:G	U:G
R	AGA	UCU	WC	U:A	
R	AGG	CCU	WC	C:G	U:G
R	CGT	ACG	WC	I:U	
R	CGC	ACG	Wob	I:C	
R	CGA	UCG	WC	U:A	I:A
R	CGG	UCG	Wob	U:G	
S	AGT	GCU	Wob	G:U	
S	AGC	GCU	WC	G:C	
S	TCT	AGA	WC	I:U	
S	TCC	AGA	Wob	I:C	
S	TCA	UGA	WC	U:A	I:A
S	TCG	CGA	WC	C:G	U:G
T	ACT	AGU	WC	I:U	
T	ACC	AGU	Wob	I:C	
T	ACA	UGU	WC	U:A	I:A
T	ACG	CGU	WC	C:G	U:G
V	GTT	AAC	WC	I:U	
V	GTC	AAC	Wob	I:C	
V	GTA	UAC	WC	U:A	I:A
V	GTG	CAC	WC	C:G	U:G
W	TGG	CCA	WC	C:G	
Y	TAT	GUA	Wob	G:U	
Y	TAC	GUA	WC	G:C	

FigS2

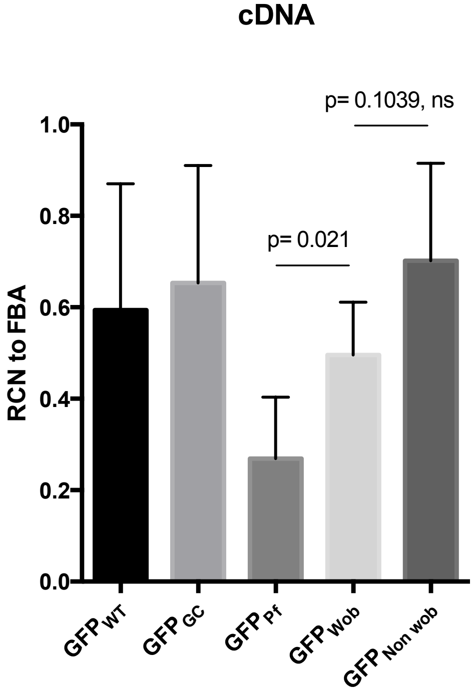

cDNA
p= 0.1039, ns
p= 0.021
RCN to FBA
1.0
0.8
0.6
0.4
0.2
0.0
GFP WT
GFP GC
GFP Pf
GFP Wob
GFP Non wob

FigS3

a

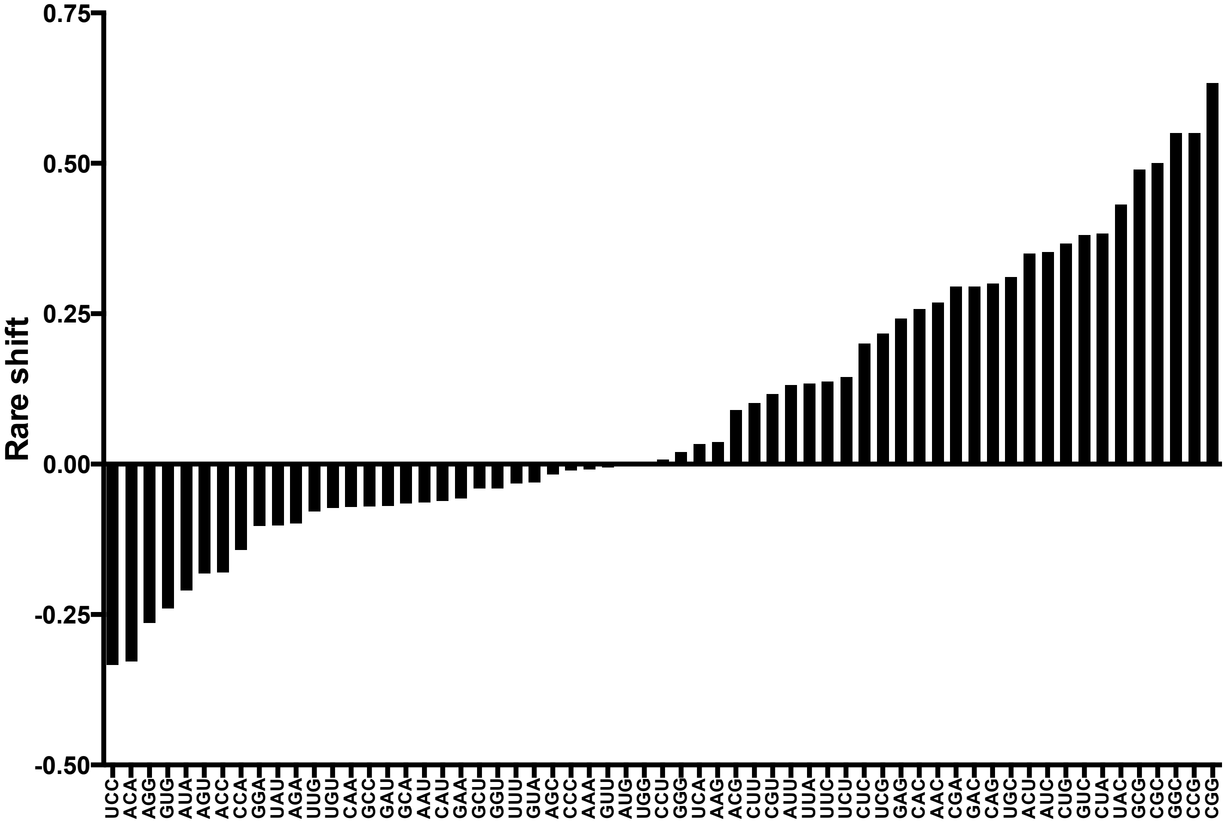
0.75
0.50
0.25
0.00
-0.25
-0.50
Rare shift
UCC ACA AGG GUG AUA AGU ACC CCA GGA UAU AGA UUG UGU CAA GCC GAU GCA AAU CAU GAA GCU GGU UUU GUA AGC CCC AAA GUU AUG UGG CCU GGG UCA AAG ACG CUU CGU AUU UUA UUC UCU CUC UCG GAG CAC AAC CGA GAC CAG UGC ACU AUC CUG GUC CUA UAC GCG CGC GGC CCG CGG

b

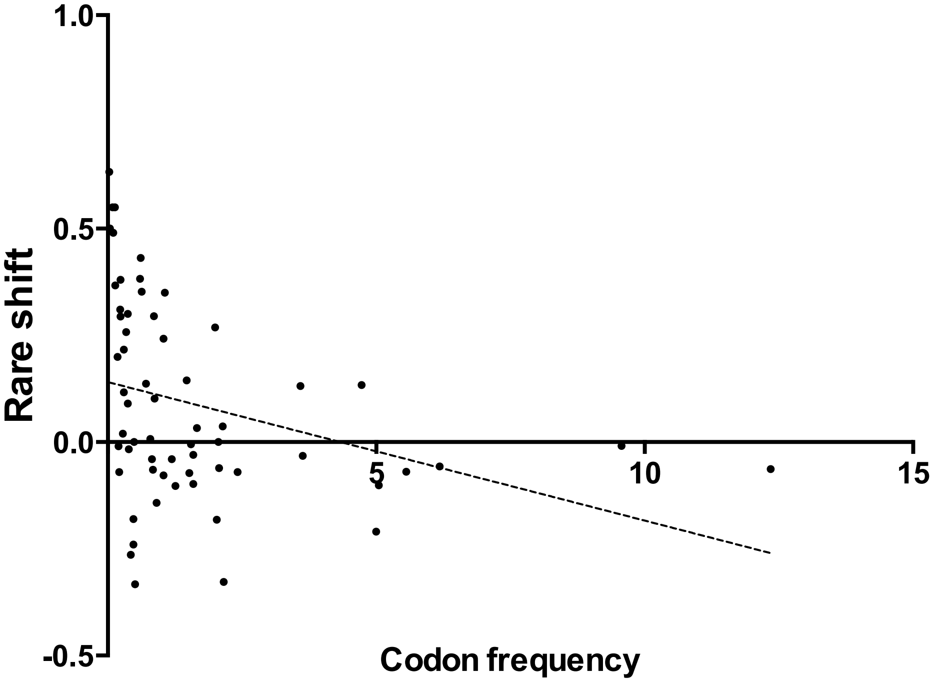
1.0
0.5
0.0
-0.5
Rare shift
5
10
15
Codon frequency

FigS4

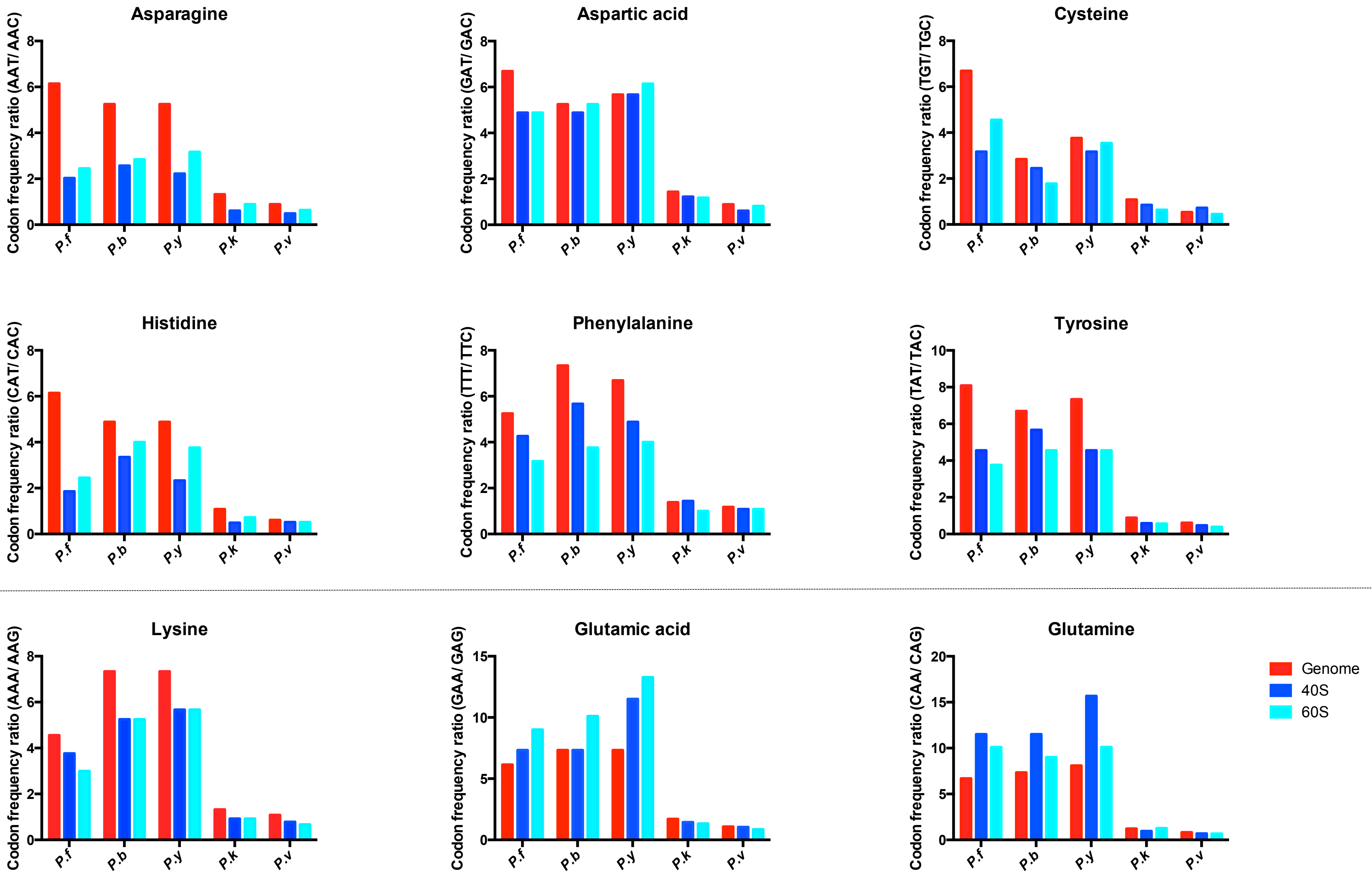

Asparagine
Codon frequency ratio (AAT/ AAC)
Aspartic acid
Codon frequency ratio (GAT/ GAC)
Cysteine
Codon frequency ratio (TGT/ TGC)
Histidine
Codon frequency ratio (CAT/ CAC)
Phenylalanine
Codon frequency ratio (TTT/ TTC)
Tyrosine
Codon frequency ratio (TAT/ TAC)
Lysine
Codon frequency ratio (AAA/ AAG)
Glutamic acid
Codon frequency ratio (GAA/ GAG)
Glutamine
Codon frequency ratio (CAA/ CAG)
P.f
P.b
P.y
P.k
P.v
Genome
40S
60S

FigS5

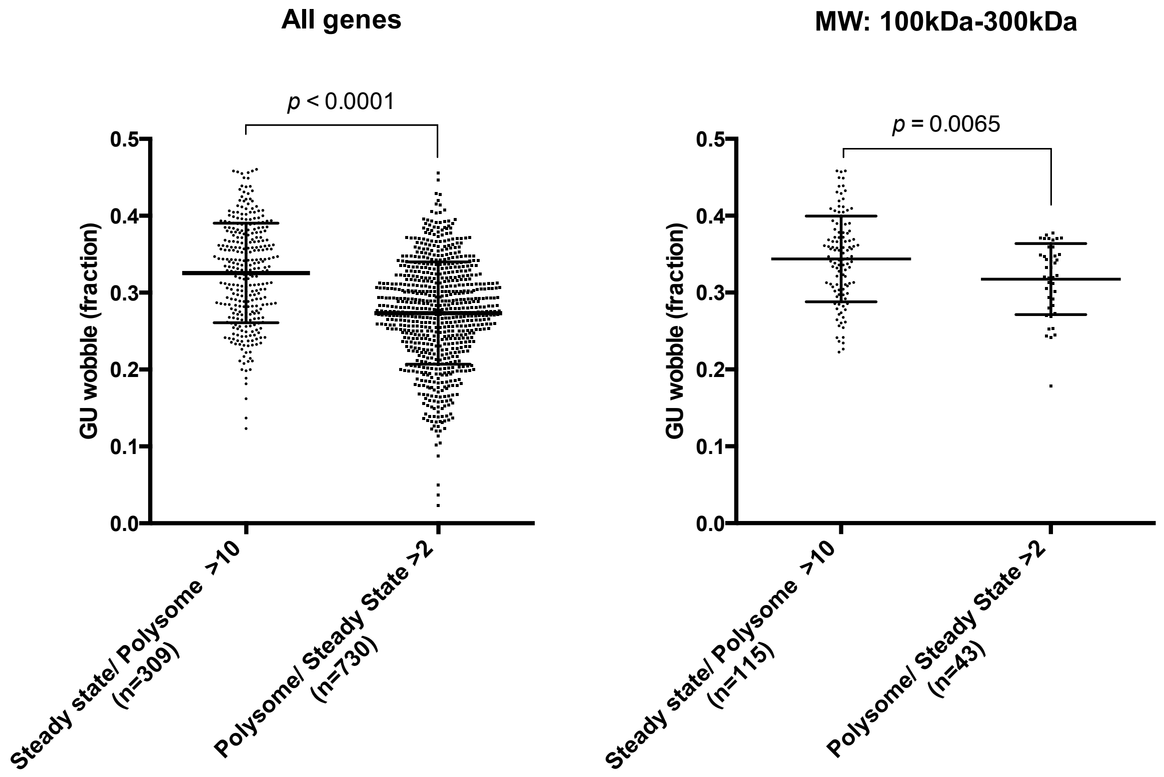
All genes
p < 0.0001
GU wobble (fraction)
0.0
0.1
0.2
0.3
0.4
0.5
Steady state/ Polysome >10 (n=309)
Polysome/ Steady State >2 (n=730)
MW: 100kDa-300kDa
p = 0.0065
GU wobble (fraction)
0.0
0.1
0.2
0.3
0.4
0.5
Steady state/ Polysome >10 (n=115)
Polysome/ Steady State >2 (n=43)

FigS6

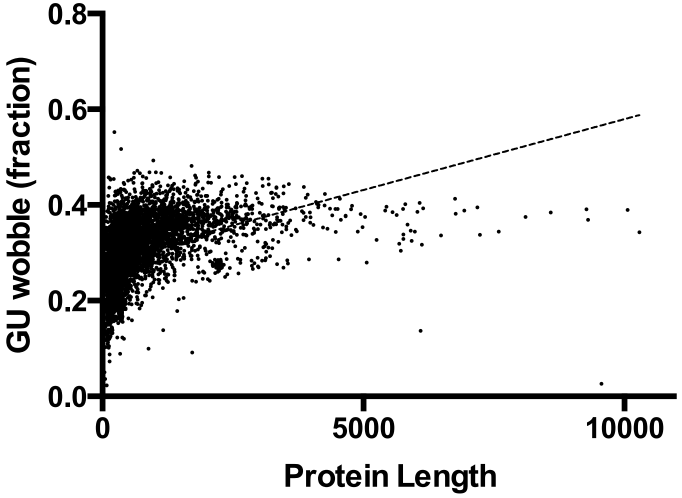

FigS7

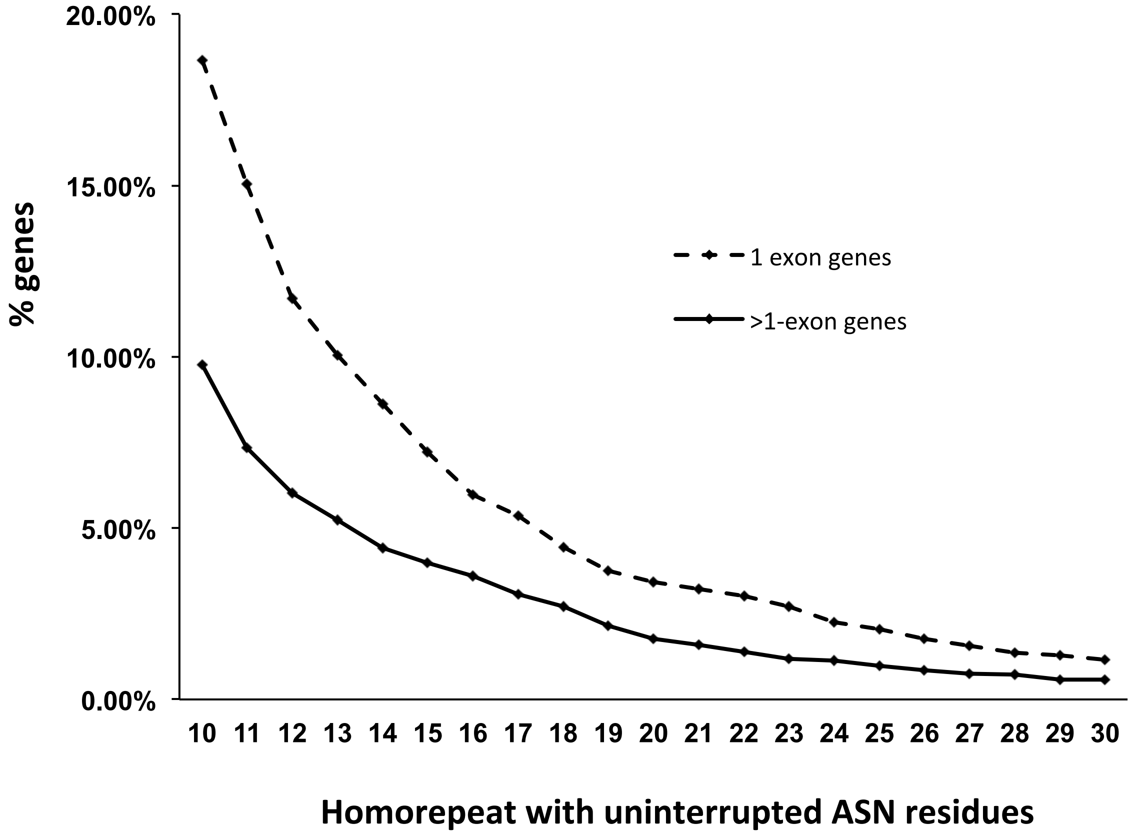
20.00%
15.00%
10.00%
5.00%
0.00%
% genes
1 exon genes
>1-exon genes
10
11
12
13
14
15
16
17
18
19
20
21
22
23
24
25
26
27
28
29
30
Homorepeat with uninterrupted ASN residues

FigS8

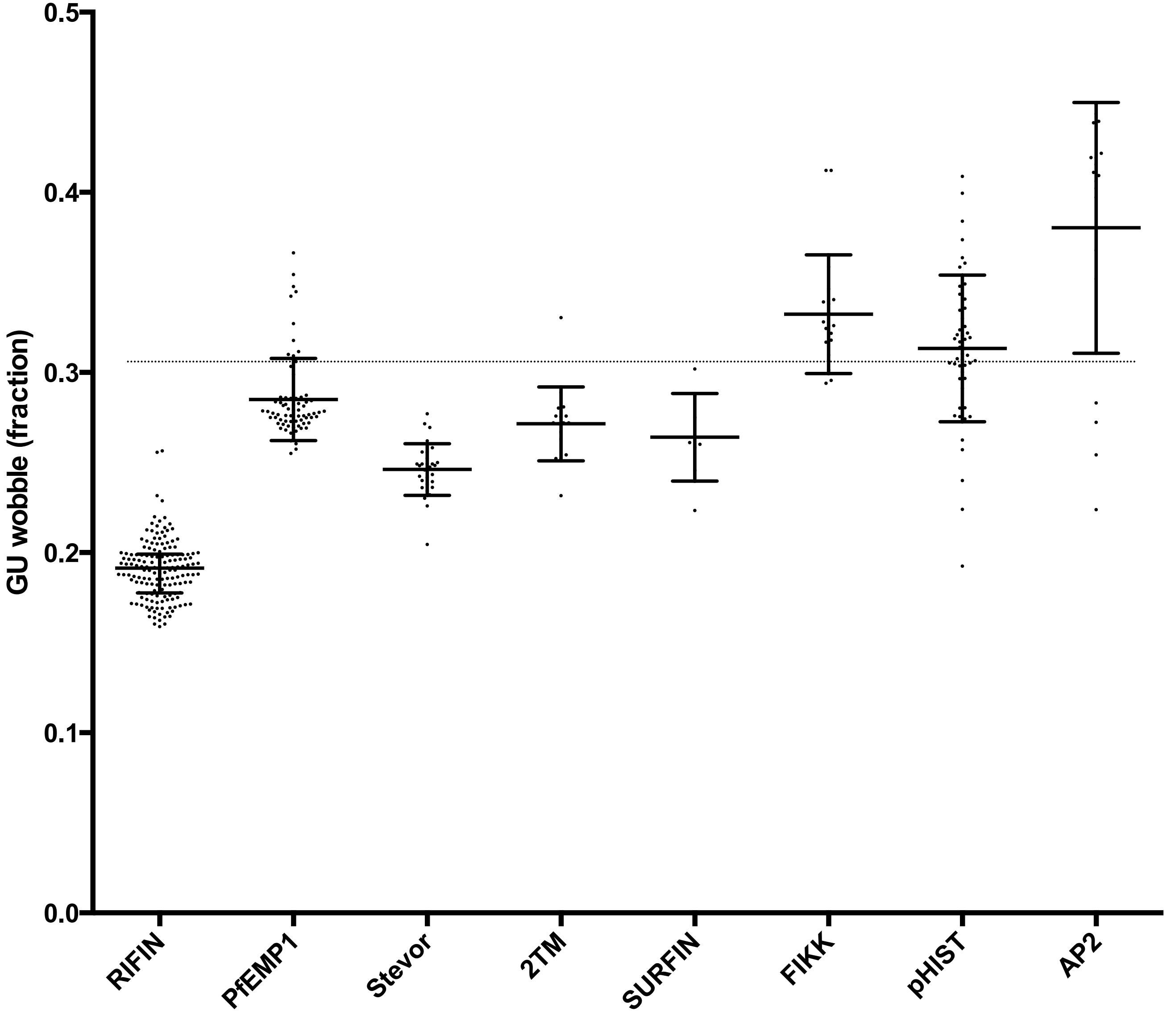

GU wobble (fraction)
0.5
0.4
0.3
0.2
0.1
0.0
RIFIN
PfEMP1
Stevor
2TM
SURFIN
FIKK
pHIST
AP2

FigS9

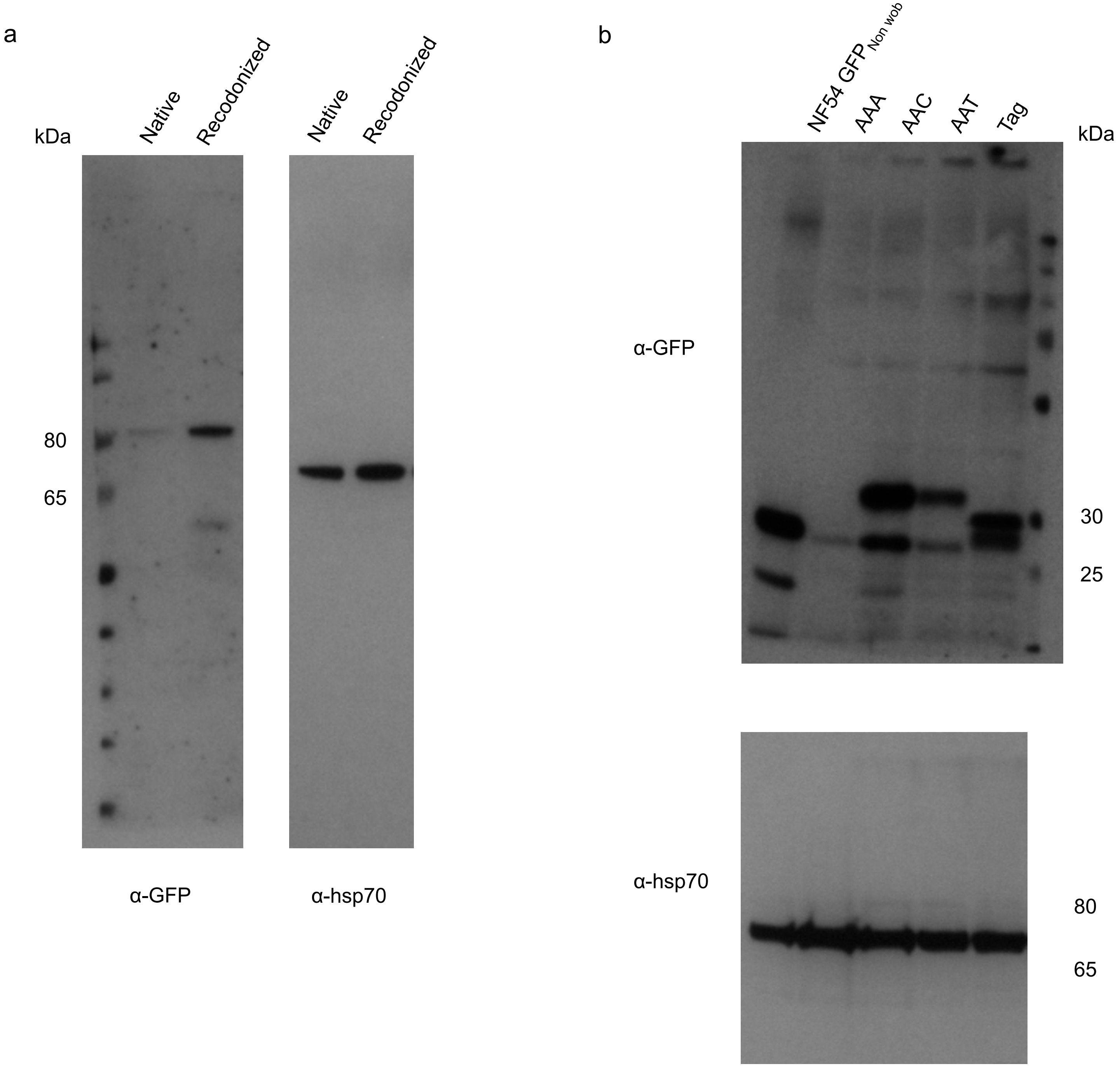
a
kDa
Native
Recodonized
Native
Recodonized
80
65
α-GFP
α-hsp70
b
NF54 GFP Non wob
AAA
AAC
AAT
Tag
kDa
α-GFP
30
25
α-hsp70
80
65
