# Supplementary material for: Frequent GU wobble pairings reduce translation efficiency in Plasmodium falciparum
Source: Sci Rep. 2017 Apr 7;7:723. doi: 10.1038/s41598-017-00801-9 (PMC5429705; doi:10.1038/s41598-017-00801-9)
Supplement: Supplementary file 3 — Supplementary info [file 41598_2017_801_MOESM3_ESM.doc]

>GFP-GC

ATGAGTAAAGGCGAGGAGCTGTTCACCGGTGTGGTGCCGATCCTCGTCGAGCTGGACGGTGACGTAAACGGGCACAAGTTCAGCGTGTCGGGCGAGGGAGAAGGCGATGCGACCTACGGTAAGTTGACGCTCAAGTTTATTTGCACAACCGGCAAGCTTCCAGTCCCGTGGCCCACGCTGGTGACCACCTTCGCCTACGGCTTACAGTGCTTTGCACGCTACCCGGACCACATGAAGCAGCACGACTTCTTCAAGTCCGCGATGCCTGAGGGGTACGTTCAGGAGCGTACGATCTTTTTCAAGGACGACGGCAACTACAAGACTCGCGCAGAAGTGAAATTCGAGGGTGATACTCTGGTTAACCGAATCGAGCTCAAGGGCATTGACTTTAAGGAGGATGGGAATATCCTGGGCCATAAGCTCGAGTATAACTACAACAGCCACAATGTGTACATCATGGCGGACAAGCAAAAAAACGGAATTAAGGTCAACTTCAAGATACGGCACAACATCGAGGATGGCTCTGTGCAGTTGGCTGACCACTACCAGCAGAATACGCCGATTGGGGATGGCCCAGTACTGCTTCCGGACAACCACTATCTGAGCACGCAATCCGCCCTATCGAAAGACCCCAACGAGAAGCGCGACCACATGGTGTTGCTGGAGTTTGTCACGGCGGCCGGCATCACACATGGAATGGACGAACTCTACAAGTAA

>GFP-Pf

ATGAGTAAAGGAGAAGAATTATTTACCGGTGTAGTTCCAATATTAGTAGAATTGGATGGAGATGTTAATGGTCATAAATTTAGTGTATCAGGAGAAGGTGAAGGGGATGCAACTTATGGAAAATTAACATTAAAATTTATTTGTACCACTGGTAAACTTCCAGTTCCTTGGCCAACATTAGTAACAACTTTTGCTTATGGGTTACAATGTTTTGCAAGATATCCTGATCATATGAAGCAACATGATTTCTTTAAATCTGCTATGCCAGAAGGATATGTGCAAGAGAGAACAATATTTTTTAAAGACGATGGTAATTATAAAACGAGAGCAGAAGTTAAGTTTGAAGGAGATACTTTGGTAAATCGTATTGAATTAAAAGGCATAGACTTCAAAGAAGATGGTAATATTTTAGGACACAAACTAGAGTATAACTACAATTCACATAATGTTTATATCATGGCAGATAAACAAAAAAATGGCATAAAAGTCAATTTTAAGATTAGACATAACATAGAAGATGGATCTGTACAATTAGCTGATCATTATCAGCAAAATACACCCATAGGTGATGGACCAGTTCTTTTACCAGATAATCATTATTTGTCAACTCAAAGTGCCTTATCCAAAGATCCTAATGAAAAACGAGATCACATGGTATTACTTGAATTTGTTACAGCAGCTGGTATTACACATGGAATGGACGAATTATATAAATAA

>GFP-Wobble

ATGAGTAAAGGTGAAGAATTATTTACCGGTGTAGTTCCAATATTAGTAGAATTGGATGGTGATGTTAATGGTCATAAATTTAGTGTATCAGGTGAAGGTGAAGGGGATGCAACTTATGGTAAATTAACATTAAAATTTATTTGTACCACTGGTAAACTTCCAGTTCCTTGGCCAACATTAGTAACAACTTTTGCTTATGGGTTACAATGTTTTGCAAGATATCCTGATCATATGAAGCAACATGATTTTTTTAAATCTGCTATGCCAGAAGGTTATGTGCAAGAGAGAACAATATTTTTTAAAGATGATGGTAATTATAAAACGAGAGCAGAAGTTAAGTTTGAAGGTGATACTTTGGTAAATCGTATTGAATTAAAAGGGATAGATTTTAAAGAAGATGGTAATATTTTAGGTCATAAACTAGAGTATAATTATAATTCACATAATGTTTATATCATGGCAGATAAACAAAAAAATGGTATAAAAGTCAATTTTAAGATTAGACATAATATAGAAGATGGTTCTGTACAATTAGCTGATCATTATCAGCAAAATACACCCATAGGTGATGGTCCAGTTCTTTTACCAGATAATCATTATTTGTCAACTCAAAGTGCCTTATCCAAAGATCCTAATGAAAAACGAGATCATATGGTATTACTTGAATTTGTTACAGCAGCTGGTATTACACATGGTATGGATGAATTATATAAATAA

>GFP-Non wobble

ATGAGTAAAGGAGAAGAATTATTCACCGGTGTAGTTCCAATATTAGTAGAATTGGACGGAGACGTTAACGGACACAAATTCAGCGTATCAGGAGAAGGAGAAGGCGACGCAACATACGGAAAATTAACTTTAAAATTCATTTGCACAACTGGAAAACTTCCAGTTCCTTGGCCAACATTAGTAACTACTTTCGCTTACGGCTTACAATGCTTCGCAAGATACCCTGACCACATGAAGCAACATGACTTCTTCAAATCTGCTATGCCAGAAGGATACGTGCAAGAGAGAACAATATTCTTCAAAGACGACGGAAACTACAAAACAAGAGCAGAAGTTAAGTTCGAAGGAGACACATTGGTAAACCGTATTGAATTAAAAGGCATAGACTTCAAAGAAGACGGAAACATTTTAGGACACAAACTAGAATACAACTACAACTCACACAACGTTTACATAATGGCAGACAAACAAAAAAACGGAATAAAAGTAAACTTCAAGATTAGACACAACATAGAAGACGGATCTGTACAATTAGCTGACCACTACCAGCAAAACACACCAATAGGAGACGGACCAGTTCTTTTACCAGACAACCACTACTTGTCAACTCAAAGCGCGTTATCTAAAGACCCTAACGAAAAACGAGACCACATGGTATTACTTGAGTTCGTTACAGCAGCTGGAATTACACACGGAATGGACGAATTATACAAATAA

>Recodonized PF3D7_0202400 sequence (amino acid residue 757-1192)

ATGGACGACGACTACGATGAATCCCTGAAAACCAAGAACTACTACCCGCACAATATGACGTTCGGTCAGCAGCAGTATTTCCCGTACTATAACCCTCTGGAGCAACAGAATTATCAGCTGCACCACATCATCAAACAGCAACAAAACTACCACCCTCATCATATCATCAAGCAACAGCAAAATCACAACCCGCATCATATTCTGCAAGAACAAGAAAAACACCACCCGCAGGGTATTCCGAAAGAACAACCGTACAATAATGTCCCGTACATTTTGAAGAAGGGCCTGGAACCGAAAACCCATAATCATGTTAAAGAGGATCAACCAAACATTAAACAAGGTGTCGTTAAGGGCCAGGAGCCTCACGTGGACGATATGCATAACAATACGAAGGAACACAAGAACTTTAAGAATACTACGGACGTCAAACAGCCGGCGAGCCACATCTATAACAACTCCAGCGAGAAACAGATTGAGCATGTTTACAATAAGAGCCCGGAAAAGCAAATTGAGCACGTGTACAACAAAAGCCCAGAGAAACAAATTGAACATGTCTACAATAACTCCCCAGAGAAGCAGATTGAACACGTCTACAACAACAGCCCGGAGAAGCAAATCGAGCATGTGTATAACAATAGCCCTGAAAAACAGATCGAACACGTTTACAACAACTCCCCGGAGAAACCGGCACGTCACACGAATAACATTAGCTTGGAAAAGCAGAATAGCCATAAGTACAACGTGAATATCCAGGACCGCCACGATCCGGTGTACTATAAGTATGAGGATATGCTGAAGCGTGACAAGGACCTGTTTACGATCATTAACAATATCTGTGAGTTGGAATTCAACAGCACCAACAACTATCTGATGAAAATCATCAACAATGACAAACTGAAACACAACAGCCTGAATGATAACGAAGCGATTCTGAAAGAGATCACCAAGACCCAGAATGAGCTGTTCAGCCTGAAGCTGCCGCTGGAGATCAAAGTGAGCATGGCGCTGCGTATCAGCGAACGTCTGCGTGCCTTTGTTTTTGACAAAGATTTGACCGCCTACTATATCAAAAAGCTGAAGGATATCTTCAAGCTGGAAACGGAAGCAGCGAAGAATTACTATTACTACGTTAAATGCCAAAAGACCTTCAGCGACAAGAAACGCCTGGTGAATAATCTGGACAGCATCAAACTGTATTATGAGTCTCAGATTAACAAAAACTTTATCAGCATTCCGAAGGACAAAATTCCGACGGCTATTTACCGCATTAGCAATCTGGTCAACGATCTGATCTTCCTGTTGCCGCAGTCTAATGCGAATAAGGCACTG
